# In vitro amplification of pathogenic tau conserves disease-specific bioactive characteristics

**DOI:** 10.1007/s00401-020-02253-4

**Published:** 2021-01-01

**Authors:** Hong Xu, Mia O’Reilly, Garrett S. Gibbons, Lakshmi Changolkar, Jennifer D. McBride, Dawn M. Riddle, Bin Zhang, Anna Stieber, Jeffrey Nirschl, Soo-Jung Kim, Kevt-her Hoxha, Kurt R. Brunden, Gerard D. Schellenberg, John Q. Trojanowski, Virginia M.-Y. Lee

**Affiliations:** 1 Department of Pathology and Laboratory Medicine, Institute On Aging and Center for Neurodegenerative Disease Research, University of Pennsylvania School of Medicine, Philadelphia, PA 19104 USA; 2 Department of Pathology and Laboratory Medicine, Penn Neurodegeneration Genomics Center, University of Pennsylvania School of Medicine, Philadelphia, PA 19104 USA

**Keywords:** tauopathy, tau spreading, tau strains, in vitro seeding

## Abstract

The microtubule-associated protein tau (tau) forms hyperphosphorylated aggregates in the brains of tauopathy patients that can be pathologically and biochemically defined as distinct tau strains. Recent studies show that these tau strains exhibit strain-specific biological activities, also referred to as pathogenicities, in the tau spreading models. Currently, the specific pathogenicity of human-derived tau strains cannot be fully recapitulated by synthetic tau preformed fibrils (pffs), which are generated from recombinant tau protein. Reproducing disease-relevant tau pathology in cell and animal models necessitates the use of human brain-derived tau seeds. However, the availability of human-derived tau is extremely limited. Generation of tau variants that can mimic the pathogenicity of human-derived tau seeds would significantly extend the scale of experimental design within the field of tauopathy research. Previous studies have demonstrated that in vitro seeding reactions can amplify the beta-sheet structure of tau protein from a minute quantity of human-derived tau. However, whether the strain-specific pathogenicities of the original, human-derived tau seeds are conserved in the amplified tau strains has yet to be experimentally validated. Here, we used biochemically enriched brain-derived tau seeds from Alzheimer’s disease (AD), corticobasal degeneration (CBD) and progressive supranuclear palsy (PSP) patient brains with a modified seeding protocol to template the recruitment of recombinant 2N4R (T40) tau in vitro*.* We quantitatively interrogated efficacy of the amplification reactions and the pathogenic fidelity of the amplified material to the original tau seeds using recently developed sporadic tau spreading models. Our data suggest that different tau strains can be faithfully amplified in vitro from tau isolated from different tauopathy brains and that the amplified tau variants retain their strain-dependent pathogenic characteristics.

**Supplementary Information:**

The online version contains supplementary material available at 10.1007/s00401-020-02253-4.

## Introduction

Microtubule-associated protein tau (tau) is intrinsically disordered and highly soluble when it is not bound to microtubules. The presence of hyperphosphorylated tau inclusions in the human brain pathologically defines a group of neurodegenerative diseases collectively referred to as tauopathies. The most prevalent tauopathy, AD, affects about 10% of the population over the age of 65 [34]. In AD patients’ brains, cognitive decline and neuron death are closely associated with the increasing burden of aggregated tau filaments known as paired helical filaments (PHFs). Spatiotemporally, PHFs are found to spread throughout anatomically connected brain regions as the disease progresses. The spreading follows a stereotypical pattern beginning in the limbic system and is subsequently transmitted to the frontal temporal lobe and neocortex. Although the spatiotemporal pattern exhibits some variability, the spread of tau pathology through anatomically connected brain regions has been documented in other tauopathies as well [8, 22, 23, 40]. Experimental evidence based on cell and animal models suggests that human brain-derived tau aggregates (tau seeds) are pathogenic, inducing the aggregation and fibrillization of endogenous, normal tau [14, 15, 31]. Once induced into the pathological state, the newly formed tau aggregates recapitulate pathological features of human tau pathology including hyperphosphorylation, insolubility and cell-type specificity [14, 21, 42, 43]. These models not only advance our insight into sporadic tau pathogenesis, but could also act as reliable biosensors for differentiating the various tau strains.

Different tau aggregates (referred to here as strains) may represent tau variants that lead to distinct tauopathies with different clinical and pathological phenotypes [31]. Earlier evidence suggests that strain specificity appears to be conformation-dependent for different species of pathological tau [14]. Biochemically, tau aggregates from different tauopathies show distinct patterns of resistance to protease digestion and/or denaturation, resulting in characteristic banding patterns of tau fragments on immunoblots [31, 37]. Mass spectrometry data revealed the amino acid sequence of these digested fragments. The varied amino acid composition of the remaining fragments suggests that, in different tau strains, different regions of the pathological tau aggregates are exposed to proteolytic digestion [37]. More recently, cryo-electron microscopy (Cryo-EM) studies confirmed that tau aggregates exhibit strain-specific structural heterogeneity at the ultrastructural level by revealing the core structures of AD, CBD, Pick’s disease (PiD) pathological tau filaments [3, 4, 7, 44]. Interestingly, the structures of tau proto-filaments exhibit high uniformity within a given tauopathy, which is consistent with the strain-dependent activity observed in both sporadic cell culture and animal models of pathological tau transmission [31].

Tau preformed fibrils (pffs), assembled in vitro in the presence of polyanionic inducers, share certain pathogenic features with human-derived tau filaments and potently induce tau aggregates in some tauopathy models. Past experiments have only demonstrated tau pff-induced pathogenesis in models that overexpress aggregation-prone, mutant human tau [13, 17, 18, 21] and have failed to recapitulate these results in animal models that express full-length wild-type (WT) tau, which indicates that tau pffs lack some disease-relevant pathogenic features [12, 14, 15]. The structural differences between tau pffs and human-derived tau aggregates provide a plausible explanation for the differences in activities, yet there is no direct evidence that connects the pathogenicity of different tau strains to their distinct structural conformations.

Though previous in vitro amplification experiments showed that pathological tau can be amplified from minute amounts and in a strain-dependent manner [24, 28, 35], there is an unexplored connection between the amplified tau strains and their pathogenicities. In this study, we hypothesize that different tau strains, i.e., pathological tau species that adopt different conformations, can template conformation-dependent tau fibrillization by recruiting tau monomers and that, as a result, the strain-specific pathogenic features are conferred to and conserved in the fibrillized product. We standardized the single-cycle in vitro amplification reactions using human-derived tau strains from AD, CBD, and PSP patients’ brains as seeds and T40 as the substrate. The seeding process yielded AD-like (ADT40P1), CBD-like (CBDT40P1), and PSP-like (PSPT40P1) pathological tau filaments, which were biochemically analyzed to confirm their strain specificity. The pathogenicity of ADT40P1, CBDT40P1, and PSPT40P1 was quantitatively assessed in vitro using WT mouse hippocampal neuron cultures as well as in vivo using mouse models that express WT tau [16].

## Materials and methods

### Animals

CD1 and C57BL6 wild type (WT) mice were purchased from Charles River. 5xFAD mice were purchased from Jackson Lab and bred with the B6/SJL F1 line for heterozygous offspring. 6hTau mice were generated by cross-breeding PAC-tau mouse line with PAC-E10 + 14  mouse line as described [27, 45]. All animal care and experimental protocols were approved by the University of Pennsylvania’s Institutional Animal Care and Use Committee.

### Purification of tau seeds

AD, CBD and PSP-tau were sequentially extracted from different regions of patient brains as previously described [14]. All cases were screened to exclude the comorbidities of Lewy body and TDP-43 pathologies based on immunohistochemical staining (data not shown). The ELISA and immunoblot measurements of α-synuclein, amyloid beta and TDP43 protein concentrations are shown in Table 2. The whole middle frontal cortex except motor cortex was used for cortical extractions, while the posterior and middle temporal lobe was used for temporal lobe extractions. For AD cases, grey matter and white matter were separated using a surgical blade and only grey matter was used in the later extractions. Brain homogenate was prepared in nine volumes (v/w, ml/g) of PHF buffer (10 mM Tris, 10% sucrose, 0.8 M NaCl, 1 mM EDTA, pH 7.4) with 0.1% sarkosyl, proteinase inhibitor and phosphatase inhibitor in a glass dounce homogenizer and spun at 10,000 g for 10 min at 4 °C. Supernatant (sup 1) was collected, sarkosyl was added to 1% final concentration, incubated in a beaker with stirring for 1.5 h at RT, and followed by 150,000 g spin for 75 min at 4 °C. The pellet was collected, briefly washed with PBS to clean the myelin, and resuspended in PBS (pel 1). To remove the sarkosyl, pel 1 was spun at 150,000 g for 75 min and resulting pellet was collected as pel 2. For AD cases, pel 2 was resuspended in PBS, thoroughly sonicated and spun at 10,000 g for 10 min at 4 °C. The supernatant was collected as the enriched tau seeds. For PSP and CBD cases, pel 2 was used as tau seeds due to the relatively lower tau-burden in the tissue.

### Expression and purification of recombinant tau

Recombinant tau proteins were expressed in BL21 (DE3) RIL *E. coli* cells and purified by fast protein liquid chromatography following previous descriptions [14]. The concentration of recombinant tau was determined using a nanodrop 1000 (ND-1000, Spectrophotometer). Heparin-induced tau pffs were made according to a previous protocol [14].

### In vitro seeding reactions

To deactivate proteolytic activity, tau seeds were first heated to 56 °C for 30 min in a thermocycler (PTC-200, Peltier Thermal Cycler) in the presence of a proteinase inhibitor cocktail (1% w/v pepstatin, leupeptin, TPCK, TLCK, and trypsin inhibitor, 0.1 M EDTA, in H_2_O) (1:100 v/v) and PMSF (50 mM). After the heat treatment, tau seeds were sonicated in a bath sonicator (Diagenode) for 20 min, with 30 s sonication and 30 s pause at the highest intensity. Tau seeds were mixed together with T40 to obtain a total tau concentration of 40 µM. For ADT40P1 and CBDT40P1 reactions, the ratio of tau seeds to monomer is 1:9. For ADT40P1*, the seeds/recombinant tau concentration ratio is 1:99. Next, 2 mM DTT was added into the solution. For PSPT40P1, the reaction was set up with 40 µM recombinant T40 with 0.2–2% of PSP-tau seeds depending on the tau concentration in the lysates. All the reactions were conducted in sterile phosphate-buffered saline (pH 7.4, without Mg^2+^ and Ca^2+^) in PCR tubes (PCR-02-C, Axygen Scientific) with 50–100 µl volume on a thermomixer (thermomixer C, Eppendorf) for 7 days at 37 °C if not specified. Day 0 samples were collected before agitation and kept frozen in a -80° C freezer until use.

### Sedimentation assay

The sedimentation assay was conducted by mixing 1 µl of the sample with 19 µl of PBS (0.1% sarkosyl, w/v) and spun for 30 min at 100,000 g with an ultracentrifuge (Optima, Beckman Coulter) as previously described [14]. The supernatant was removed and the pellet was suspended in 20 µl of PBS. Lysates were mixed with loading buffer for immunoblots.

### Transmission and immuno-EM

EM was conducted as described previously [14]. Negative staining of filaments for EM was performed with 2% uranyl acetate except as noted below. Immuno-EM was carried out using the AT8 antibody and the anti-myc antibody followed by anti-mouse and anti-rabbit secondary antibodies conjugated to 12 or 6 nm colloidal gold, respectively. Negative staining was performed in these experiments with 1% uranyl acetate. EM images were taken with a JEOL 1010 EM instrument. T40 monomers were treated with the same protocol to control for nonspecific staining. Quantification of EM was performed by ImageJ software.

### Primary neuron cultures and transduction of tau fibrils

CD1 mouse cortices and hippocampi were dissected at embryo day 16–18 and dissociated with papain (Worthington Biochemical Corporation). Neurons were resuspended in neural basal medium (Gibco, 21,103) with 2% B27 (Gibco), 1 × Glutamax (Gibco) and 1 × penicillin/streptomycin (Gibco). Plates or coverslips were coated with poly-d-lysine (0.1 mg/ml, Sigma-Aldrich) in borate buffer (0.05 M boric acid, pH 8.5) overnight at room temperature. Cells were plated at a density of 50,000 cells/cm^2^ for all types of plates. When plating, 5% FBS was added to the cell suspension. The plating medium was replaced with neural basal medium without FBS at day 1 in vitro (DIV1). At DIV7, the plating medium was replaced with conditioned medium containing 1:1 ratio of old and fresh medium. Tau fibrils were diluted in PBS to the desired concentrations, added on top of the cells and incubated for 14 days until DIV 21. The dose of tau fibrils was scaled based on cell densities (μg tau/10^6^ cells) per different plate. For activity testing, 1 μg tau/10^6^ cells was used for 100% seeds (100% pathogenic tau), 0.1 μg tau/10^6^ cells for 10% seeds control and 0.01 μg tau/10^6^ cells for 1% seeds control unless otherwise specified. All the amplified tau variant doses were based on the total tau concentration at the start of the in vitro reaction. For PSP seed controls, the seeds were diluted to match the reaction conditions and transduced onto the neurons. To compare the ultracentrifuge fractionated pellet of the amplified tau variants to the seeds, the tau concentrations were first estimated using immunoblots (Fig. 3c) and then transduced on neurons with the same dose.

### Immunocytochemistry

At DIV 21, cells in 24 well plates with coverslips were washed with PBS once and fixed with ice-cold methanol for 20 min in a − 20 °C freezer. For 96 and 384 well plates, cells were washed four times with PBS in a plate washer (BIOTEK), extracted with 1% hexadecyltrimethylammonium bromide (HDTA, Sigma-Aldrich) for 10 min and fixed for 10 min with 4% paraformaldehyde and sucrose in PBS. Fixed cells were stained with primary antibodies overnight at 4 °C and Alexa Fluor-conjugated secondary antibodies (Thermo Fisher Scientific) for 2 h at room temperature. Photomicrographs were obtained with a light microscope (Eclipse Ni, Nikon) and quantified using HALO software as described previously [14]. Confocal photomicrographs were taken with a Leica DMI 6000 microscope coupled with a white-light laser.

### Stereotaxic injection of pathogenic tau fibrils

Mice were anesthetized with ketamine/xylazine/acepromazine and fixed on a stereotaxic frame (David Kopf Instruments). 5xFAD and WT mice were unilaterally double injected with tau fibrils at bregma: − 2.5 mm, lateral: + 2 mm, depth: − 2.4 mm and − 1.4 mm from the skull at right dorsal hippocampus and cortex. Two micrograms of tau was injected for the 100% seeds controls (2 μg AD-tau) and ADT40P1 (0.2 μg AD-tau + 1.8 μg T40 with agitation for 7 days) or 10% seeds control (0.2 μg of AD-tau + 1.8 μg T40 without agitation). 6hTau mice were bilaterally injected (bregma: − 2.5 mm, lateral: ± 2 mm, depth: − 2.4 mm from the skull). The right hemisphere was used for histology. The left hemisphere was used for biochemistry.

### Extraction of insoluble tau and immunoblots

Cells were washed once with PBS and harvested in PBS containing 1% sarkosyl and proteinase inhibitor cocktail. The lysates were incubated on ice for 15 min and then spun at 100,000 g for 30 min at 4 °C. The supernatant was kept as the soluble fraction, and the pellet was suspended in PBS as the insoluble fraction. For mouse brain tissues, hippocampi were dissected after perfusion with PBS at a flow rate of 1.5 ml/min for 20 min and homogenized in nine volumes of PHF extraction buffer containing 1% Triton-X100. Lysates were spun at 100,000 g for 30 min at 4 °C. The supernatant was collected as Triton-soluble fraction (TX-S), and the pellet was suspended in distilled water containing 1% sarkosyl, incubated at room temperature for 1 h and then spun at 100,000 g for 30 min at 4 °C. The supernatant was collected as the sarkosyl-soluble fraction (Sark-S) and the pellet saved as the sarkosyl-insoluble fraction (Sark-P). Fifteen micrograms of total proteins from TX-S and Sark-S was loaded on the immunoblots. Sark-P was loaded at a 10:1 ratio to the respective TX-S fractions. The immunoblots were generated as previously described [13]. Primary antibodies were incubated overnight at 4 °C followed by secondary incubation of 2 h at room temperature. Photomicrographs were taken using an imager (Li-Cor Biosciences). The optical densities were measured using ImageJ Software. For dot blot studies, AD-tau, ADT40P1 and T40 samples were diluted to the desired concentrations in Tris-buffered saline (TBS) and loaded on 0.4 mm PVDF membranes and blocked with 5% milk. Primary and secondary antibodies (Thermo Fisher Scientific) were incubated following the same protocol as for immunoblots.

### Heatmaps of tau pathology

Heatmaps of the distribution of AT-8 tau pathology were generated as previously described [15]. Each region of the brain was scored for the presence of tau pathology between 0 and 3, given 0 is completely free of tau pathology and 3 is the most abundant tau pathology. The semiquantitative scores of AT-8 tau pathology were obtained in a blinded manner.

### Histology and immunofluorescence (IF) staining

Mouse brains were dissected after perfusion with PBS at a flow rate of 1.5 ml/min for 20 min. The brains were immersion fixed with 10% neutral buffered formalin (NBF) and processed following a previously described protocol [15]. Six-micrometer-thick brain sections were stained with different primary antibodies and developed using a polymer horseradish peroxidase detection kit (BioGenex). For immunofluorescence, the brain sections were incubated with 3R (RD3) and 4R (4Rtau) tau-specific antibodies overnight at 4 °C followed by a 2-h incubation of Alexa Fluor-conjugated secondary antibody (Thermo Fisher Scientific). Autofluorescence was quenched using a 0.3% Sudan black solution.

### Proteinase K digestion

ADT40P1, CBDT40P1 and PSPT40P1 pellets were fractionated using an ultracentrifuge at 100,000 g for 30 min and suspended in PBS. Tau concentrations were estimated using immunoblots. 0.5 μg of tau variants was mixed with 0.0005% (w/v) proteinase K in PBS buffer and incubated at 37 °C for 30 min. The reactions were stopped by boiling the samples at 100 °C with 1 × loading buffer for 10 min. The same concentrations of T40 and hepT40 were used as controls.

### WND-CHARM analysis of tau pathology

Supervised classification was performed for testing of the morphological differences of tau pathologies induced by different tau strains. Top 15% of the images features were used for training the classifier. For each time of the test, a random set of 70% of the images were used to train the classifier to predict AD vs PSP vs CBD using the image features. The other 30% of the images were used for the classification. The test was performed 100 times independently. Twenty-five percent of downsample was used in the analysis. Command line is: wndchrm test -f0.15 -r#0.70 -n100 -d25 -N3 –mls. Images from four biological repeats were used for the analysis.

### Antibody dilution

All antibodies were diluted according to the supplementary table, online resource.

### Statistical analyses

Unpaired *t* tests were performed to compare two experimental groups. For comparisons of more than two groups, one-way ANOVA and two-way ANOVA was performed, followed by Tukey post hoc test. All the statistics were performed using Prism software (GraphPad Software, Inc). Statistical significance was reached when *p* ≤ 0.05. If not specified, data are presented as the mean ± standard deviation.

## Results

### AD-tau exhibits consistent biochemical properties and pathogenic activity

**Fig. 1:**
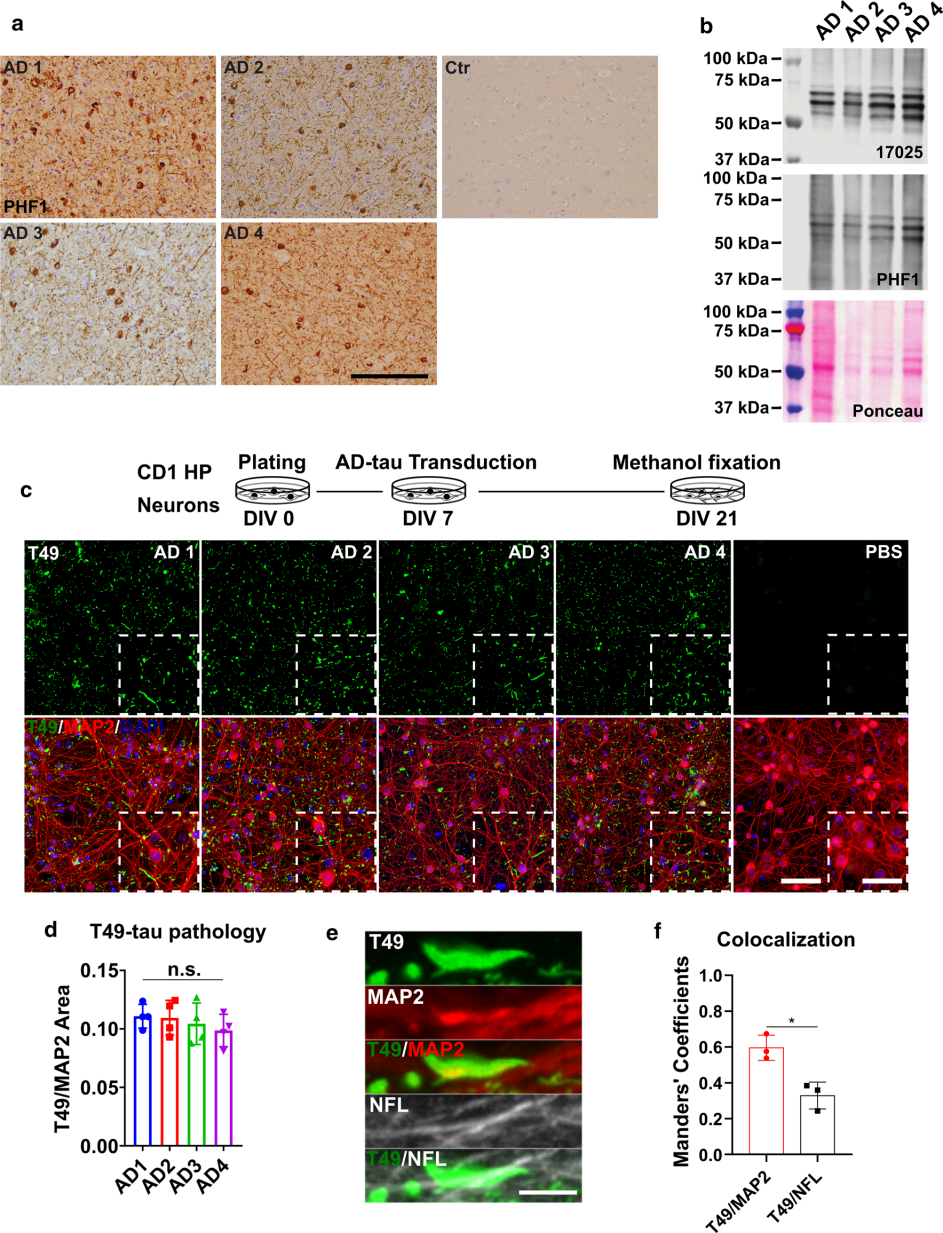
Insoluble tau from different AD cases exhibits consistent seeding abilities in WT mouse primary neurons. **a** Immunohistochemistry (IHC) of frontal cortex sections from different AD patient brains and non-tau pathology control using the PHF1 anti-phosphorylated-tau antibody; scale bar = 500 µm. **b** Immunoblot of sarkosyl-insoluble AD-tau probed with a total tau (17,025) and a phospho-tau (PHF1) antibody; ponceau staining shows total protein in the samples. **c** Immunocytochemistry (ICC) of AD-tau-induced mouse tau pathology in CD1 mouse primary hippocampal neuron cultures revealed by a mouse tau-specific antibody (T49) with concomitant staining of cell nuclei (DAPI) and neuronal dendrites (MAP2 antibody); scale bar = 100 µm and 25 µm (inset). The schematic diagram shows the experimental paradigm for the neuron culture-based activity assay. **d** Quantification of T49-mouse tau pathology from **c**. T49-postive area was normalized to the MAP2 positive area; *n.s.* nonsignificant for all groups; one-way ANOVA followed by Tukey post hoc test; *n* = 4. **e** Representative images of T49-positive tau pathology shows the relative location of AD-tau induced mouse tau pathology in dendrites (MAP2) and axons (NFL); scale bar = 5 µm. **f** Colocalization of T49/MAP2 and T49/NFL from **e**; *n* = 3

To standardize the in vitro reaction system for AD-tau amplification, we first focused on AD-tau due to its relative abundance. AD-tau was enriched by sequential extraction from the brains of AD patients following a previously described protocol [14]. To enrich AD-tau, we specifically chose brains with relatively short postmortem intervals and high tau-burdens at late stages of the disease course (Table 1). Immunohistochemical staining using a phospho-tau antibody confirmed that all four cases had abundant neuropil tau pathology as well as neurofibrillary tangles (NFTs) (Fig. 1a).

**Table 1:** AD, CBD, and PSP tau seeds used in the study

	Insoluble Tau	Tau/Total protein	Brain region	Tau score	Braak stage	Disease Duration (yr)	PMT (hr)	Age (yr)	Sex
AD1	1.70 µg/ul	13%	Mid frontal cortex	3+	VI	10	12	62	M
AD2	1.80 µg/ul	27%	Mid temporal lobe	3+	VI	8	9	68	F
AD3	3.30 µg/ul	28%	Mid frontal cortex	3+	VI	9	4	73	F
AD4	1.00 µg/ul	15%	Mid frontal cortex	3+	VI	12	4	71	M
CBD1	0.25 µg/ul	7.60%	Mid frontal cortex	3+	0	5	9	59	F
CBD2	0.27 µg/ul	13%	Mid frontal cortex	3+	II	4	6	63	M
PSP1	0.0004 ug/ul	0.08%	Mid frontal cortex	3+	0	5	16	72	M
PSP2	0.37 µg/ul	9%	Mid frontal cortex	3+	II	5	7	63	F
PSP3	0.061 µg/ul	0.70%	Mid frontal cortex	3+	0	5	14	72	F
Control	N.D	N.A	Mid frontal cortex	0	0	N.A	16	62	M

*N.D.* not detectable, *N.A.* not available

To eliminate the possibility that variation in our experimental results could be due to intrinsic variability between the four selected cases, we first sought to demonstrate that the AD-tau extracted from each case did not exhibit significant variations in their biochemical properties and bioactivities. Immunoblot analysis showed the same number of major bands across all four cases, indicating that the extractions yielded the same tau species (Fig. 1b). To assess the bioactivity of the AD-tau, we adopted a previously reported neuron culture-based assay [14] that can sensitively differentiate tau pathology induced by different human-derived tau strains, including AD, CBD, and PSP tau strains, and does not respond to heparin-induced tau pffs (hep-pffs), ensuring that any observed pathology is the consequence of the added human pathogenic tau material [31]. The AD-tau enriched from all four AD cases induced comparable amounts of insoluble mouse tau pathology, as revealed by T49, a mouse tau-specific antibody. Additionally, the morphologies of the mouse tau pathology appeared as dot-like and linear structures identical to the neuritic tau pathology seen in human AD brains. The pathology primarily manifested as neuritic tau aggregates with a relatively homogenous distribution (Fig. 1c, d, and e). When the AD-tau preparations were used at the concentration of 1 μg tau/10^6^ cells, there was no sign of neurotoxicity based on the morphology of the neuronal perikarya and their processes revealed by MAP2 staining (Fig. 1c). The absence of neurotoxicity validated that pathology could be induced without compromising cell viability. Immunocytochemistry demonstrated that pathological tau aggregates were often found to be colocalized with MAP2-positive dendrites and less frequently with NFL positive axons (Fig. 1f). This finding is consistent with previous observations that, under pathological conditions, tau mislocalized from axonal to somato-dendritic compartments [2, 26, 33]. Overall, these data indicate that the AD-tau extracted from different late-stage AD brains were highly consistent in their biochemical and biological properties.

### ADT40P1 is pathogenic

**Fig. 2:**
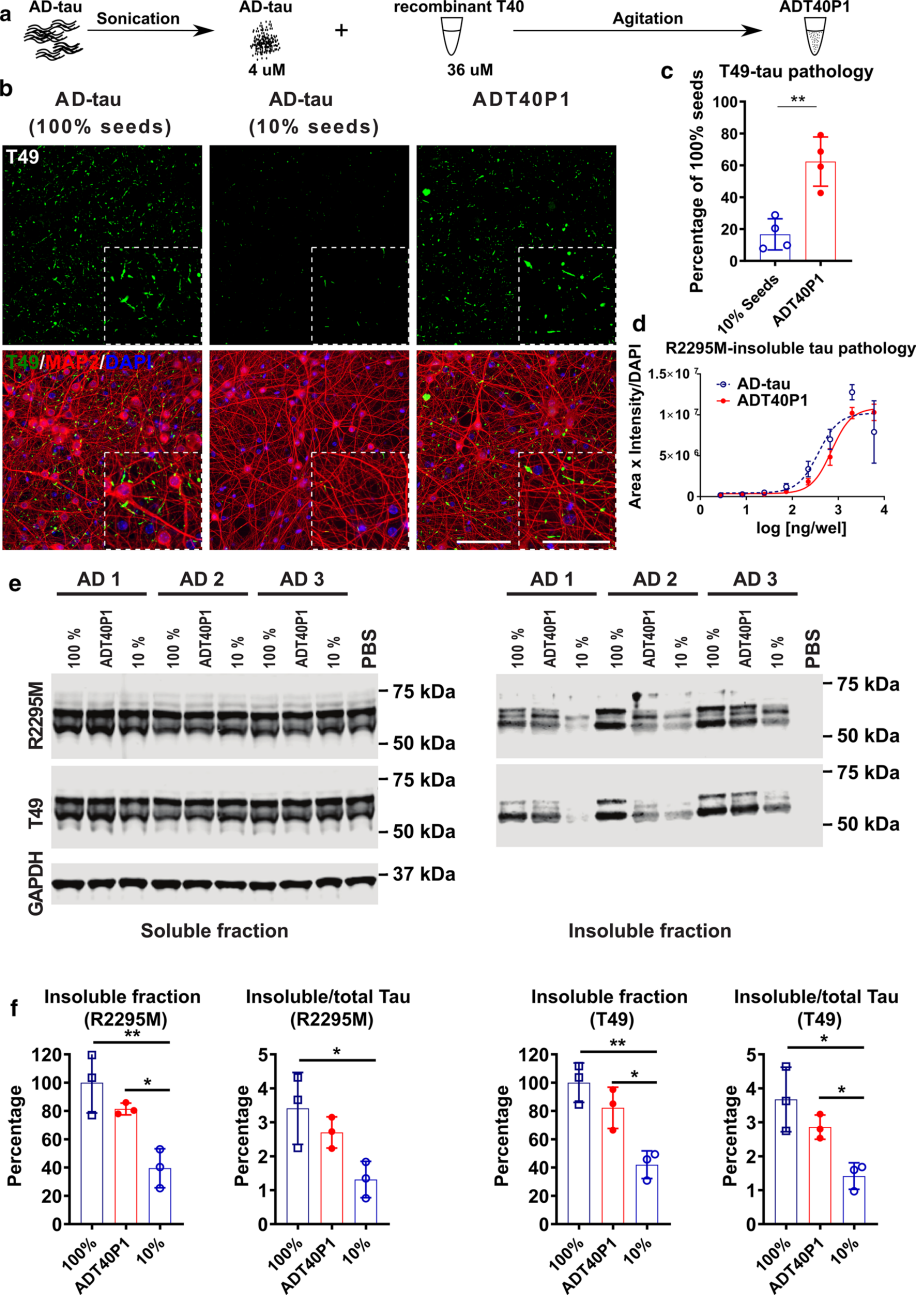
ADT40P1 exhibited pathogenicity in WT neuron culture. **a** Schematic representation of the steps necessary to generate ADT40P1. **b** ICC of AD-tau or ADT40P1-induced mouse tau pathology visualized using a mouse tau-specific antibody (R2295M) on WT mouse neuron cultures; 100% seeds represents the maximum amount of pathology induced by ADT40P1 if 100% of T40 is fibrillized during amplification; 10% seeds represents the minimum amount of pathology induced by the seeds alone if no T40 is fibrillized during amplification; scale bar = 100 µm and 25 µm (inset). **c** Quantification of T49-positive tau pathology from **b**; T49-positive area was normalized to MAP2 area; pathological % of tau seeding activity was set as 100%; **P < 0.01 unpaired t test; *n* = 4. **d** Dose–response curve of ADT40P1- and AD-tau-induced mouse tau pathology in WT neuron cultures visualized using a mouse tau-specific antibody (R2295M); R2295M area was multiplied by intensity and then normalized to DAPI counts; EC50: AD-tau = 368.4; ADT40P1 = 714.1; *n* = 4. **e** Immunoblots of soluble and insoluble tau fractions extracted from the WT neurons treated with ADT40P1, 100% AD-tau seeds, or 10% AD-tau seeds. Blots were probed with two mouse tau-specific antibodies (T49 and R2295M), while GAPDH was used as an internal loading control. **f** Optical density quantification of **e**; normalized to 100% AD-tau control; *P < 0.05, **P < 0.01, one-way ANOVA followed by Tukey post hoc test; *n* = 3

To amplify AD-tau in the in vitro seeding reactions, we chose T40, the 2N4R recombinant tau isoform, as our substrate because it contains all the subdomains of tau protein observed in the mature human brain. We designated the resulting product, AD-tau seeded recombinant T40 passage 1, as ADT40P1. To accomplish the amplification, we use in vitro reactions generated by agitating a mixture containing 10% (w/w) sonicated AD-tau + 90% T40 to induce formation of ADT40P1 (Fig. 2a). The AD-tau extracts contained a significant amount of protein contaminants that showed strong proteolytic activities (Table 1, tau/total protein ratio), which resulted in the degradation of the T40 substrate in the initial reactions (Supplementary Fig. 1a, online resource). To reduce proteolysis, we modified our protocol by adding proteinase inhibitors (PI) into the reactions, which effectively reduced the degradation of T40 in the reaction mixtures and permitted the seeding reaction to occur and resulted in increased insolubility of T40 as revealed by sedimentation assay (Supplementary Fig. 1a and b, online resource). To quantitatively analyze the biological activity of the seeded ADT40P1, we used AD-tau as controls in the neuron culture assay. The 100% seed control represents the maximum possible yield of ADT40P1 when 100% of the T40 substrate was fibrillized into pathogenic tau filaments during the seeding reaction. We also used a baseline control containing only 10% AD-tau seeds and no T40 to represent the minimum amount of pathology induced by the seeds alone if no amplification during the seeding reaction.

In the neuron culture assay, ADT40P1 showed a threefold increase in activity, as measured by the total mouse tau pathology, compared to the 10% seeds control (Fig. 2b and c, Supplementary Fig. 1c and d, online resource), indicating that the human-derived tau seeds induced substantial formation of pathogenic tau during the amplification reaction. The activity of ADT40P1 was, on average, 60% of that of the 100% AD-tau seeds control, which indicates that not all of the T40 was converted into pathogenic AD-tau. Neither agitated 10% seeds nor T40 monomer resulted in any increased activity following agitation compared to the non-agitated seeds control (Supplementary Fig. 1c and d, online resource), suggesting that both AD-tau seeds and T40 substrate must be present for the seeding reaction to occur. While the presence of a protease inhibitors was necessary to prevent the degradation of T40 thereby promoting the seeding reaction, it did not cause the fibrillization of T40 or increase the activity of the seeds. Additionally, we did not observe any signs of neurotoxicity as a result of the addition of proteinase inhibitors (Supplementary Fig. 2, online resource).

Full dose–response curves, based on the hexadecyltrimethylammonium bromide (HDTA)-stable mouse tau pathology, showed that ADT40P1 activity reaches the same plateau as AD-tau in the neuronal assay (Fig. 2d), indicating that the pathogenic tau species in ADT40P1 is equally potent to AD-tau. As an additional quantitative assessment of ADT40P1 activity, we measured the level of sarkosyl-insoluble tau in the treated neurons using biochemical fractionation and immunoblots. We found a consistent and significant increase in insoluble tau induced by ADT40P1 compared to the 10% AD-tau seeds control as revealed by two different mouse tau-specific antibodies, T49 and R2295M. There was no significant change in the soluble tau levels (Fig. 2e and f).

To demonstrate that the increased activity of ADT40P1 compared to the 10% seed control is due to specific seeding of AD-tau, rather than other insoluble material in the lysates, we performed additional control reactions using non-AD brain lysates that contained the same amount of total insoluble protein as the 10% seed control. We did not observe any tau pathology induced by these seeded samples in the neuronal assay, indicating that T40 is not seeded by other insoluble components in the brain lysate (Supplementary Fig. 3, online resource). To demonstrate the specificity of the neuronal assay to AD-tau, we tested the activity of heparin-induced tau pffs (hep-T40) as well. These hep-T40 pffs, when used at the same dose as AD-tau, did not show any bioactivity in the assay (Supplementary Fig. 3, online resource), confirming the specificity of the assay in responding to human-derived tau strains.

**Fig. 3:**
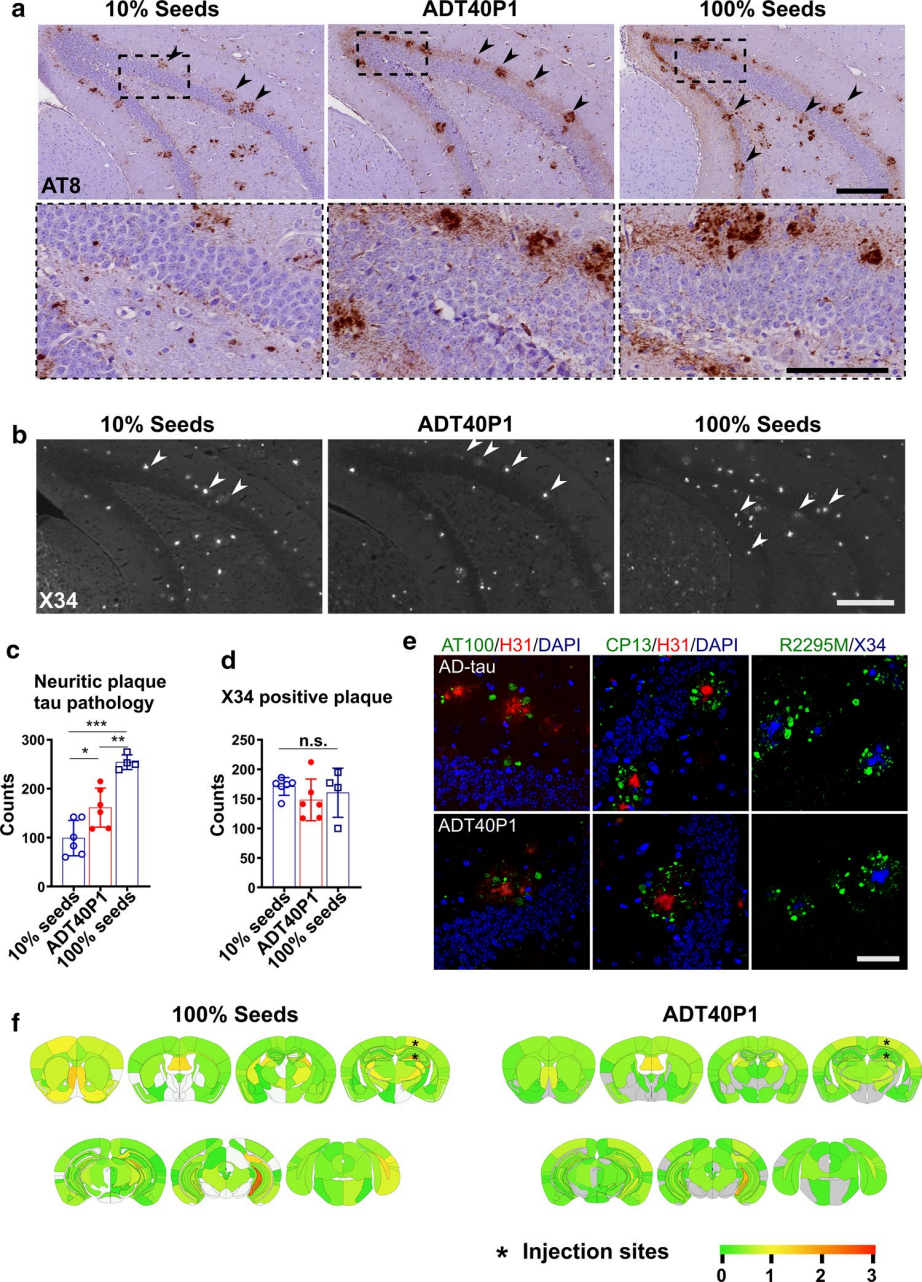
ADT40P1 is pathogenic in vivo. **a** IHC of 6-month-old 5xFAD mouse brain sections. Mice injected with the same concentration of tau for 100% seeds control (100% AD-tau), 10% seeds control (10% AD-tau + 90% T40, no agitation) and ADT40P1. Mice were killed at 1-month post-injection and brains were probed with the AT8 antibody for phospho-tau; upper panel scale bar = 200 µm; low panel scale bar = 100 µm; arrow heads show amyloid-beta plaque adjacent to neuritic plaque tau pathology. **b** X34 staining of sections adjacent to those in **a**; arrow heads show neuritic plaque tau pathology associated amyloid-beta plaques in stained by the amyloid histochemical dye X34; scale bar = 200 µm. **c** Quantification of AT8 tau pathology from **a**; *P < 0.05 10% seeds vs ADT40P1, **P < 0.01 ADT40P1 vs 100% seeds, ***P < 0.001 100% seeds vs 10% seeds, one-way ANOVA followed by Tukey post hoc test; *n* = 6 for 10% seeds and ADT40P1 groups, *n* = 4 for 100% seeds control. **d** Quantification of X34-positive amyloid-beta plaques from **b**; n.s. nonsignificant, one-way ANOVA followed by Tukey post hoc test; *n* = 6 for 10% seeds and ADT40P1 groups, *n* = 4 for 100% seeds control. **e** Immunofluorescence staining (IF) of neuritic plaque tau pathology visualized using phosphor-tau (AT100/CP13) and mouse tau antibodies (R2295M) together with anti-amyloid beta (1–42) H31L21 antibody and X34 dye; scale bar = 50 µm. **f** Heat map of AT8-positive pathology distribution from **a**; 100% AD seeds vs ADT40P1. Gray represents no pathology. Low to high tau pathology score is represented as a gradient of green to red hues

To assess the activity of ADT40P1 in vivo, we first used the well-characterized 5xFAD mouse model [32], which overexpresses human mutant APP and PSEN1, leading to the accumulation of AD-like Aβ plaque pathology with age. Our prior studies showed that, following intracerebral injection of AD-tau, the 5xFAD mice developed neuritic tau pathology primarily surrounding the Aβ plaques [15]. Consistent with the neuron culture assay, we used 100% AD-tau seeds control to reflect maximum fibrillization. For the 10% seed control, we matched the total dose of injected tau by mixing 10% AD-tau + 90% T40 without agitation to control for the amount of pathology that could be induced by AD-tau without exogenously seeding the reaction. Neuritic plaque tau pathology was analyzed in paraffin-embedded brain sections using a phospho-tau antibody (AT8). At the 1-month post-injection interval, we found morphologically identical neuritic plaques induced by ADT40P1 and AD-tau (Fig. 3a). The adjacent sections stained with X34, an amyloid-binding dye preferentially detect mature forms of amyloid fibrils [19, 25]. The dye revealed that amyloid plaque cores are closely associated with AT8-positive neuritic plaque tau pathology (Fig. 3b). These results are consistent with our previous report on the induction of neuritic tau pathology following intracerebral AD-tau injections in aged 5xFAD mice [15]. This association was further confirmed by double-label immunofluorescence staining using additional phospho-specific (AT100/CP13), mouse tau-specific antibodies (R2295M), the H31L21 antibody (H31, specific for Abeta 1–42) or the X34 dye (Fig. 3e). We found a significant increase in the tau-positive neuritic plaque burden in the ADT40P1 injected mice compared to 10% seed control group (Fig. 3a and c), indicating that the increased activity of ADT40P1 can be recapitulated in vivo. On average, the tau pathology induced by ADT40P1 accounted for 50% of that of 100% seed control, consistent with our observations in the neuron culture assay. By injecting the same dose of hep-T40 alone, we confirmed that the observed tau pathology in 5xFAD mice was specific to AD-tau and ADT40P1 (Supplementary Fig. 4, online resource). Quantification of X34-positive Aβ plaques showed no significant difference between the groups (Fig. 3d), indicating that the difference in neuritic plaque tau pathology was not due to the variation in Aβ plaque pathology. Heat maps generated by semiquantitative analyses of AT8 tau pathology showed a comparable spreading pattern of the tau pathology following injection of AD-tau or ADT40P1 (Fig. 3f), which supports the observation that tau pathology spreads in a consistent, spatial–temporal pattern.

WT mice do not overexpress tau or any disease-related transgenes and exhibit a high threshold of pathogenesis [14]. In WT mice, endogenous mouse tau has only been observed to interact with human brain-derived tau seeds to develop human-like tau pathology specific to the strain properties of exogenous tau seeds [31]. Their ability to recapitulate human disease pathology makes WT mice an ideal model for demonstrating AD tau-specific pathogenicity of ADT40P1. To quantitatively assess the bioactivity of ADT40P1 in WT mice, we compared intracerebral injections of ADT40P1 to 100% and 10% seed controls (Supplementary Fig. 5a, online resource). At 6-month post-injection, we quantified the amount of tau pathology in paraffin-embedded brain sections using the AT8 antibody. Consistent with our hypothesis, the mice injected with ADT40P1 showed an increased amount of tau pathology in both the ipsilateral and contralateral caudal hilus and the ipsilateral entorhinal cortex compared to the 10% seeds cohort (Supplementary Fig. 5a, online resource). The increased tau pathology is validated by two quantification measures: the area occupancy of AT8-positive immunoreactivity as well as a total count of NFTs present in these regions (Supplementary Fig. 5b, online resource). A semiquantitative heat map of total brain pathology demonstrates that the 100% seeds control and ADT40P1-injected mice develop pathology with a similar spatial–temporal distribution (Supplementary Fig. 5c, online resource).

To further interrogate the seeding effects of AD-tau, we evaluated their potency by reducing the concentration of AD-tau seeds in the reactions to only one tenth of that in the previous reactions. These new preparations contain 1% AD-tau and 99% of T40 to yield ADT40P1*. The average activity of ADT40P1* is significantly higher than 1% seed control in vitro and in vivo (Supplementary Fig. 6a–g, online resource). Following agitation, we observed elongated, fibrillar structures in the ADT40P1* preparations (Supplementary Fig. 6b, online resource). The ADT40P1* data validate the efficacy of the seeding reaction, demonstrating that a reduced percentage of AD-tau seeds can still recruit T40 monomers to form pathogenic fibrils, which are capable of inducing tau pathology both in vitro and in vivo.

Altogether, our data support the hypothesis that AD-tau seeds can recruit T40 monomers to form pathogenic tau. These tau species are potent both in vitro and in vivo.

### ADT40P1 recapitulates the biochemical and pathogenic features of AD-tau

**Fig. 4:**
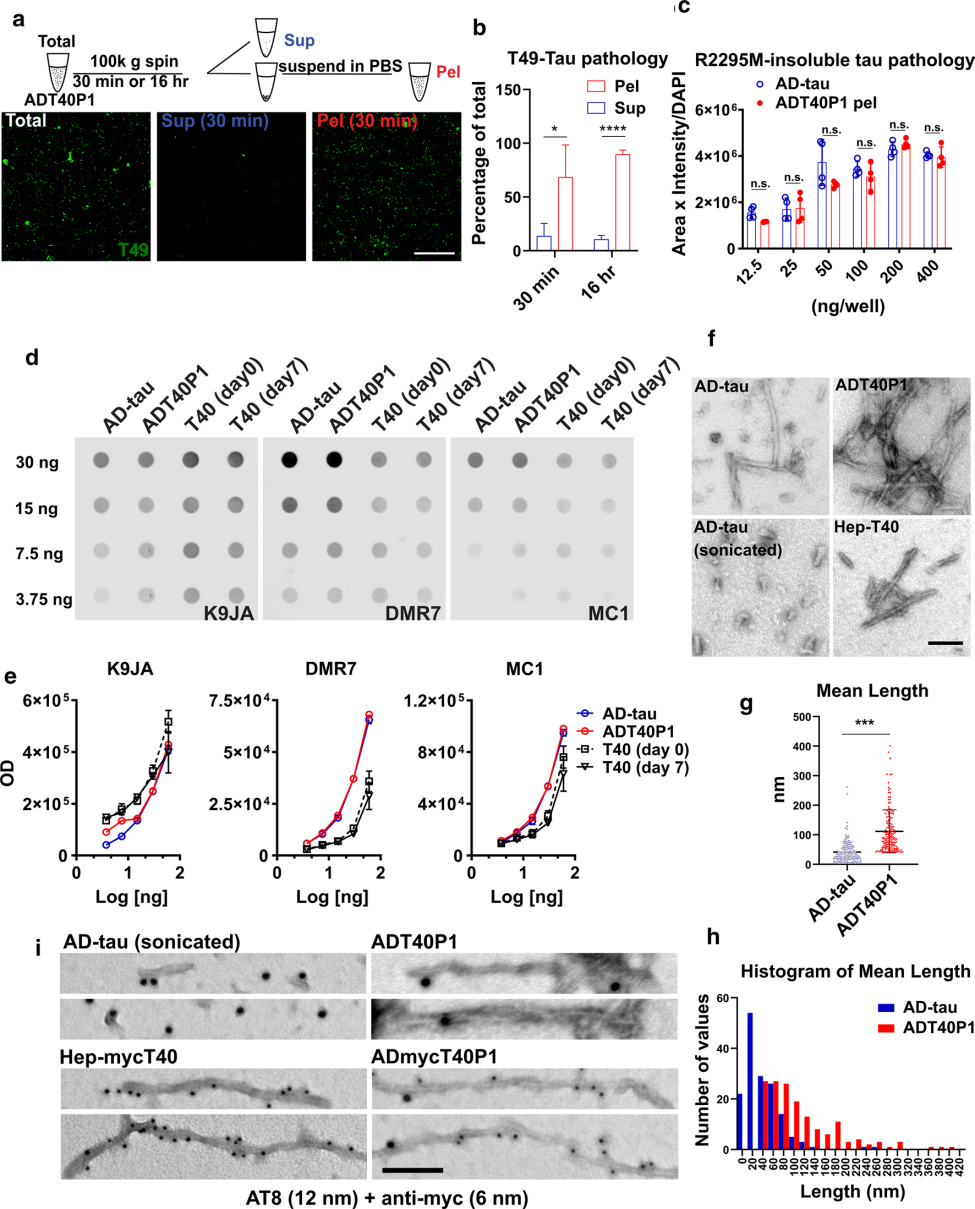
ADT40P1 shares biochemical features with AD-tau. **a** IHC of WT neurons treated with biochemically separated soluble and insoluble ADT40P1; mouse tau pathology was visualized using T49 antibody; sup = supernatant, pel = pellet; scale bar = 100 µm. **b** Quantification of T49 tau pathology from **a**; T49-positive area was normalized to MAP2 area; total ADT40P1 activity level was set as 100%; *P < 0.05, ***P < 0.001, unpaired *t* test; *n* = 4. **c** Quantification of R2295M-positive tau pathology induced by AD-tau and pellet fraction of ADT40P1 at 6 different doses in neuron culture model; R2295M-positive area was normalized to MAP2 area; n.s. nonsignificant; two-way ANOVA followed by Tukey post hoc test; *n* = 4. **d** Dot blot of ADT40P1 and controls probed for total tau (K9JA pAb) and pathological the conformation of tau (DMR7 and MC1 antibodies). **e** Optical density quantification of **d**; n = 4. **f** Transmission EM of AD-tau (before and after sonication)-, ADT40P1- and heparin-induced tau pffs (hep-T40); scale bar = 100 nm. **g** Quantification of the length of filaments from **f**; ***P < 0.001, unpaired t test; n = 156. **h** Histogram of length of filaments shown in **g**. **i** Immuno-EM of ADT40P1, AD-tau, ADmycT40P1 (AD-tau seeded myc-T40), and hep-mycT40 (heparin-induced mycT40 pffs) using AT8 (12 nm colloidal gold beads) and anti-myc (6 nm colloidal gold beads) antibodies; scale bar = 50 nm

Previous reports have documented that pathogenic tau species are insoluble aggregates, while relatively soluble tau species are low in potency [31]. Tau insolubility, therefore, is a proxy for the seeding activity of pathological tau in human tauopathies. To determine whether ADT40P1 recapitulates the insolubility of the AD-tau seeds, we used ultracentrifugation to fractionate ADT40P1 for 30 min or 16 h. The activities of both the supernatants and pellets were assessed using the neuron culture assay. We found that 68% and 89% of the pathogenic material was recovered in the 30 min and 16 h pellet fractions, respectively (Fig. 4a and b). This suggests that the potent tau species in ADT40P1, like human-derived AD-tau, are relatively insoluble. Additionally, we estimated the tau concentration in the pellet fraction of ADT40P1 using immunoblots and found that the tau concentration in the pelleted fraction of ADT40P1 was approximately two to threefold higher than the initial amount of tau in the AD-tau seeds, confirming that the seeding reaction increased the quantity of active material in ADT40P1. After removing the soluble tau from ADT40P1 by ultracentrifugation, the pelleted fractions of ADT40P1 (ADT40P1 pel) exhibited comparable activity to the same concentration of AD-tau (Fig. 4c). The activity of ADT40P1 pel confirms that the potency of the amplified insoluble tau is very close to the potency human-derived AD-tau.

To determine whether ADT40P1 reflected the pathological conformations of AD-tau, we used two, well-characterized conformational-specific tau antibodies (DMR7 and MC1) raised to AD-tau [10, 11, 20, 41]. On dot blots, we observed a similar level of K9JA immunoreactivity across all samples, indicating equal amounts of total tau loaded on the blots. On the same blots, DMR7 and MC1 preferentially bind to ADT40P1 and AD-tau compared to T40, indicating that ADT40P1 possesses the same pathological conformations as AD-tau while T40 does not (Fig. 4d and e). As an additional control, we measured the affinity of DMR7 and MC1 for T40 that had undergone the same agitation protocol used in the seeding reaction to exclude the potential effect of agitation on T40 conformations. The data suggest that ADT40P1 shares the pathological conformation of AD-tau.

To further characterize the ultrastructure of ADT40P1, we used transmission EM to determine whether ADT40P1 shares the filamentous ultrastructure of AD-tau. The amplified material in ADT40P1 exhibited elongated, fibrillar structures similar to unsonicated AD-tau (Fig. 4f). The average length of the filaments, as estimated by EM, was significantly different between the sonicated AD-tau seeds and ADT40P1 (Fig. 4g and h), suggesting that the AD-tau recruited T40 and assembled into elongated filaments during the reaction. In the absence of AD-tau seeds, no T40 fibrils were observed after 7 days of agitation (data not shown). To confirm that the T40 monomers are incorporated in the elongated fibrils observed in ADT40P1, we set up seeding reactions with a myc-tagged version of T40 and performed double immunostaining of the ADmycT40P1 product using the AT8 (phospho-tau) and anti-myc antibodies. Immuno-EM confirmed that the tau proteins in ADmycT40P1 were filamentous and positive for phospho-tau and myc-tag (Fig. 4i). To confirm the specificity of the double staining, AT8 immunoreactivity was detected in AD-tau and ADT40P1 but not in heparin-induced myc-tagged T40 pffs (Hep-mycT40). Similarly, myc-tag immunoreactivity was found in hep-mycT40 in the absence of phospho-tau signals. Only ADmycT40P1 filaments were positive for both antibodies (Fig. 4i). Additionally, positive phospho-tau immunoreactivity was mostly found on fibril ends, while the mycT40 was distributed throughout the entire fibrils. The antibody localization indicates a fibrillization mechanism by which human AD-tau seeds recruit T40 monomers at both ends of the fibrils with exposed beta-sheet structures, which we interpret to be the attachment sites for the monomers. These data validate our hypothesis that T40 monomers are recruited by AD-tau seeds and are converted into fibrillar tau structures. The pathological conformation of elongated, fibrillar structures observed in ADT40P1 correlates with the increased activity in cell culture assay, thereby demonstrating a connection between the pathological properties of ADT40P1 and its fibrillary ultrastructure.

**Fig. 5:**
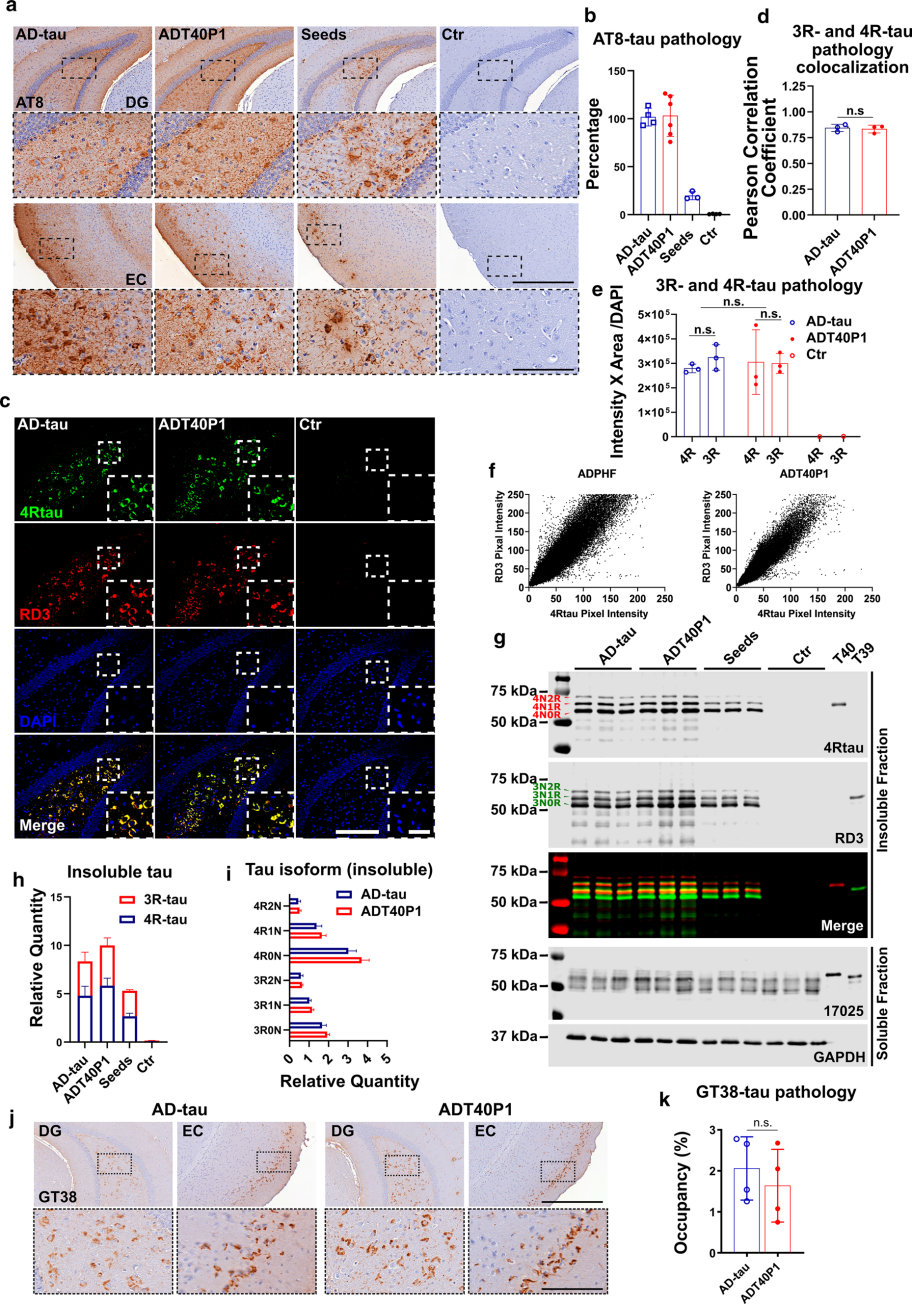
ADT40P1 and AD-tau recruit 3R and 4R tau isoforms in vivo. **a** IHC of 6hTau mouse brains injected with AD-tau or ADT40P1 at 3-month post-injection; Seed controls were used to demonstrate the amount of tau pathology induced by AD-tau in the ADT40P1 reaction mixture; controls (Ctr) show noninjected age-matched 6hTau mice; AT8 was used to visualize hypophosphorylated tau aggregates; representative images show the ipsilateral dentate gyrus (DG) and entorhinal cortex (EC); Scale bar = 500 µm and 125 µm (inset). **b** Quantification of AT8 tau pathology from **a**; AD-tau was set as 100%; n.s. nonsignificant AD-tau vs ADT40P1; one-way ANOVA followed by Tukey post hoc test; *n* ≥ 3. **c** IF of sections of 6hTau mouse brains injected with AD-tau or ADT40P1 in the hippocampus at 3-month post-injection time; Brain sections were costained for 3R- (RD3) and 4R-tau (4Rtau) isoforms; Scale bar = 200 µm and 50 µm (inset). **d** Colocalization of 3R- and 4R-tau immunoreactivity from **c**; n.s. nonsignificant; unpaired *t* test; *n* = 3. **e** Quantification of 3R- and 4R-tau pathology from **c**; n.s. nonsignificant; two-way ANOVA followed by Tukey post hoc test; *n* = 3. **f** Scatter plot of 3R- and 4R-tau positive signals from **c**. **g** Immunoblots of Triton-soluble and sarkosyl-insoluble fractions of ADT40P1- and AD-tau-injected 6hTau mouse brains; blots were probed with RD3 and 4Rtau antibodies for tau isoforms, K9JA probed for total tau, and GAPDH was used as an internal control for loading error; T39 = recombinant 2N3R tau. **h** Quantification of insoluble 3R- and 4R tau from **g**; *n* = 3. **l** Quantification of each isoform of tau from **g**; *n* = 3. **j** IHC of 6hTau mouse brains injected with AD-tau or ADT40P1 at 3-month post-injection; AD-tau pathology was visualized by the conformational antibody GT38. **k** Quantification of GT38-positive tau pathology from **j**; *n* = 4; n.s. nonsignificant AD-tau vs ADT40P1; unpaired *t* test

Prior studies have shown that different pathogenic tau strains are composed of different tau isoforms [45]. AD-tau contains an approximate 1:1 ratio of 3R and 4R tau isoforms, while PSP and CBD-tau filaments are comprised primarily of 4R tau isoforms and PiD-tau filaments are comprised primarily of 3R tau isoforms. The variation in isoform composition may be due to unique bioactive properties of the pathogenic tau filaments associated with different tauopathies. In a previous study, we injected 6hTau mice, which overexpress all six human tau isoforms, with AD-tau and observed that the mice develop pathological tau aggregates that are composed of both 3R and 4R tau [45]. The use of T40 as the monomeric substrate in the amplification reaction biased the ratio of ADT40P1 toward the 2N4R isoform (Fig. 1b, Supplementary Fig. 1a, online resource). Therefore, we sought to determine whether the tau isoform recruitment of ADT40P1 would be affected by its skewed 3R:4R ratio. We performed intracerebral injections with either AD-tau or ADT40P1 into 6hTau mice and waited for 3 months to allow the injected material to induce tau pathogenesis. At the 3-month post-injection interval, we assessed the 3R-to-4R ratio of the induced tau pathology by immunohistochemistry and biochemistry. To allow for a direct comparison, we adjusted the dose of AD-tau so that the amount of induced tau pathology was comparable to that of the ADT40P1-injected cohort. Immunohistochemical staining confirmed that there was a comparable amount of AT8-positive tau pathology overall in the AD-tau- and ADT40P1-injected cohorts. NFTs and neuropil thread tau pathologies were found in the caudal hilus region in both groups (Fig. 5a and b), while no tau pathology was observed in the age-matched, noninjected control group (Fig. 5a and b). We costained the mouse brain sections with the isoform-specific 4Rtau and RD3 antibodies to label 4R and 3R tau isoforms, respectively, in the induced pathological tau aggregates (Fig. 5c). In both groups, quantification analysis showed high levels of colocalization of 4R and 3R tau (Fig. 5d). Importantly, the ratio of 4R to 3R tau pathology in the injected animals is approximately 1:1 in both groups (Fig. 5e), which indicates that, despite the overrepresentation of 4R tau in ADT40P1, the active recruitment of 3R and 4R tau by AD-tau is conserved in ADT40P1.

Following sequential extraction of the hippocampi of the injected mice, we were able to confirm the presence of all three isoforms of 3R and 4R tau in the insoluble tau fraction (Sark-P) (Fig. 5g). The immunobands of the insoluble tau fractions showed higher MW than soluble tau which is indicative of their hyperphosphorylated state (Fig. 5g). Consistent with the immunohistochemistry data, quantification of the immunoblots showed comparable ratios of 3R to 4R tau isoforms, validating that each isoform was recruited by both AD-tau and ADT40P1 (Fig. 5h and i).

To further assess the similarities between AD-tau and ADP40P1, we used GT38, a conformation-dependent tau antibody that binds to the pathological tau with the presence of both 3R and 4R tau isoforms. A prior study demonstrated that GT38 detected AD-specific tau pathology in human patient brains but not 4R tau-dominant PSP, CBD, or 3R tau-dominant PiD-tau pathology [10]. Importantly, GT38 can detect tau pathology induced by AD-tau in 6hTau tau mice, while it fails to detect pathology induced by PSP-tau, CBD-tau or PiD-tau [45]. To demonstrate that the tau pathology induced by ADT40P1 is indistinguishable from that of AD-tau, we immunostained the 6hTau mouse brain sections with the GT38 antibody. We found that mouse brains injected with AD-tau and ADT40P1 exhibit comparable GT38 immunoreactivity (Fig. 5j and k), confirming that the bioactivity of AD-tau is indeed conserved in ADT40P1 in vivo.

### Strain-specific bioactivity was conserved during amplification of different tau strains

**Fig. 6:**
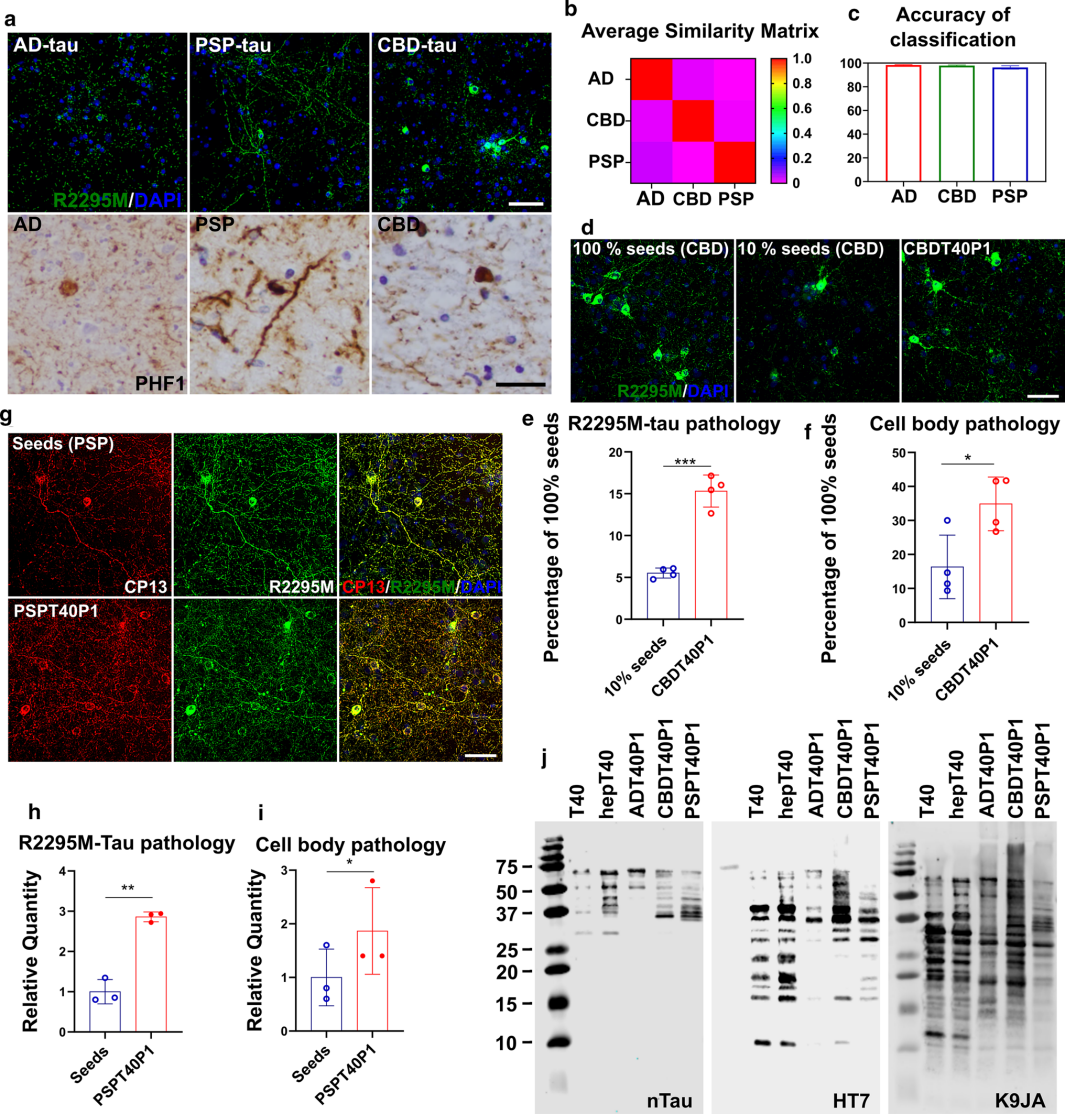
Fidelity of in vitro amplification is maintained among different tau strains. **a** Upper panel: ICC of AD-tau-, CBD-tau- or PSP-tau-treated WT mouse neurons reveals distinct patterns of mouse tau pathology visualized using a mouse tau-specific antibody (R2295M); scale bar = 50 µm; lower panel: IHC of AD, CBD, PSP patient brain sections stained with PHF1 anti-phospho-tau antibody; Scale bar = 50 µm. **b** The average similarity matrix generated by WND-CHARM software based on morphological patterns of mouse tau pathology induced by AD-tau, CBD-tau, PSP-tau in WT neurons. **c** The accuracy of pretrained WND-CHARM software in the differentiation of mouse tau pathology induced by AD-tau, CB-tau, and PSP-tau in WT mouse neurons. **d** ICC of neurons treated with CBD-tau seeded T40 (CBDT40P1) and controls; Scale bar = 50 µm. **e** Quantification of R2295M-tau pathology in **d**; ***P < 0.001; unpaired *t* test; *n* = 4. **f** Cell body pathology count of **d**; *P < 0.05; unpaired *t* test; *n* = 4. **g** ICC of neurons treated with PSP-tau seeded T40 (PSPT40P1) and controls; Scale bar = 50 µm. **h** Quantification of R2295M-tau pathology in **g**; **P < 0.01; unpaired *t* test; *n* = 3. **i** Cell body tau pathology count in **g**; *P < 0.01; unpaired t test; *n* = 3. **j** Immunoblots of proteinase K digestion products of ADT40P1, CBDT40P1, and PSPT40P1 with a C-terminal Tau antibody (K9JA), proline-rich domain tau antibody (HT7) and N-terminal Tau antibody (nTau)

Using the extraction protocol described above, we enriched CBD and PSP tau strains from human patient brains although the amount of tau recovered from these brains were overall much less than from AD brains (Table 1). Like AD-tau, the extracted CBD and PSP-tau induced endogenous mouse tau aggregation in primary WT mouse neurons. The different tau strains induce morphologically distinct tau pathology in mouse neurons (Fig. 6a upper panel). Across all the cases we have tested (Tables 1 and 2), AD-tau induced homogenously distributed dot-like and linear tau dystrophic neurites and neuropil threads with very sparse perikaryal pathology. PSP-tau induced heterogeneously distributed neuropil threads with strong perikaryal tau inclusions. CBD-tau induced a homogenous distribution of both tau dystrophic neurites with the strongest and largest numbers of neuronal perikaryal distribution of all three strains. The morphological differences observed in mouse neuron culture recapitulate neuronal tau pathologies observed in human brains probed with an anti-phospho-tau antibody (PHF1) (Fig. 6a lower panel). Using WND-CHARM software, we can reliably differentiate the three strains of induced tau pathologies based on their unique morphological signatures (Fig. 6b). With a pretrained, supervised classification, the software could correctly identify which of the three tau strains had induced mouse tau pathology in the neurons with greater than 95% accuracy (Fig. 6c). These results support the hypothesis that AD-, CBD-, and PSP-derived tau aggregates induce tau pathology in a strain-specific manner that can be reliably differentiated in mouse primary neurons by both human and machine learning.

**Table 2:** Other pathological proteins in AD, CBD and PSP seeds

	α-Synuclein	A-beta 40	A-beta 42	TDP43
AD1	0.50 ng/μl	53.70 pg/μl	60.09 pg/μl	4.29 ng/μl
AD2	1.27 ng/μl	54.53 pg/μl	279.50 pg/μl	0.26 ng/μl
AD3	0.72 ng/μl	22.32 pg/μl	102.70 pg/μl	0.44 ng/μl
AD4	0.35 ng/μl	18.14 pg/μl	73.53 pg/μl	1.80 ng/μl
CBD1	0.022 ng/μl	5.09	8.26 pg/μl	N.A
CBD2	0.028 ng/μl	N.D	6.69 pg/μl	N.A
PSP1	0.0175 ng/μl	N.D	6.55 pg/μl	N.A
PSP2	0.28 ng/μl	N.D	N.D	N.A
PSP3	0.0587 ng/μl	N.D	22.79 pg/μl	N.A
Control	1.00 ng/μl	N.D	54.15 pg/μl	N.A

*N.D.* not detectable, *N.A.* not available

Using the same fibrillization reaction that generated ADT40P1, we seeded in vitro amplification reactions with CBD and PSP tau strains to yield CBDT40P1 and PSPT40P1, respectively. To form CBDT40P1, we used the same 1:9 ratio of seeds to monomers as described above. The CBDT40P1 pathogenicity was quantitatively assessed in the neuron culture-based activity assay relative to the activity of the 10% and 100% seed controls. Like ADT40P1, CBDT40P1 shows increased activity compared to the 10% seed control, as measured by R2295M-immunolabeled mouse tau (Fig. 6d–f). Consistent with CBD-tau morphology, the perikaryal pathology also increased proportionally with overall pathology (Fig. 6d and f).

The PSP strain amplification protocol required some modifications, as the tau seeds enriched from PSP patient brains are lower in quantity and purity compared to AD-tau and CBD-tau (Table 1). Due to the limitation of reaction volume, we could not perform PSP amplification reactions with the 1:9 seeds to monomers ratio with the established amount of total tau in the reaction (40 µM). Thus, the reactions were set up with the maximum amount of PSP-tau that can be included in the reaction volume and the bioactivity of PSPT40P1 is compared to the control samples (seeds control) with the same amount of PSP-tau seeds at the starting point of the reaction. We also could not compare the amplified PSPT40P1 to the total tau dose-matched 100% seeds control because of the low yield and purity of PSP seeds. Despite these limitations, we did observe both an increase in the total mouse tau pathology in neurons treated with PSPT40P1 compared to the seeds control (Fig. 6g and h) and perinuclear pathology characteristic of PSP-induced morphology (Fig. 6g and i). The morphological signatures of ADPT40P1-, CBDT40P1- and PSPT40P1-induced tau pathology were consistent with their respective seed controls (Figs. 2b, 6d and g). These results support our hypothesis that the seeding reaction is strain-dependent and that the pathogenic characteristics of the material used to seed the reaction are conferred to and conserved in the amplified material.

The core structures of tau filaments are known to be distinct among AD, PSP, and CBD-tau [3, 7, 37]. To examine the conformational differences between ADT40P1, PSPT40P1, and CBDT40P1, we performed proteinase K (PK) digestion of the amplified materials. The digested products were analyzed by immunoblots using N-terminal (nTau), proline-rich domain and C-terminal tau-specific antibodies (Fig. 6j). The immunoblots also demonstrated that the banding pattern of the three amplified tau variants is distinct from each other, which is indicative of their conformational differences. Thus, the pathogenic characteristics and structural conformations of the amplified materials manifest strain specificities, indicating that, during the in vitro amplification process, the tau seeds recruited T40 monomers and confer strain-specific ultrastructure and pathogenic properties to the newly formed fibrils, which further induces morphologically distinct tauopathy in neuron culture.

### CBDT40P1 and PSPT40P1 recapitulate strain-specific pathogenicity in vivo

**Fig. 7:**
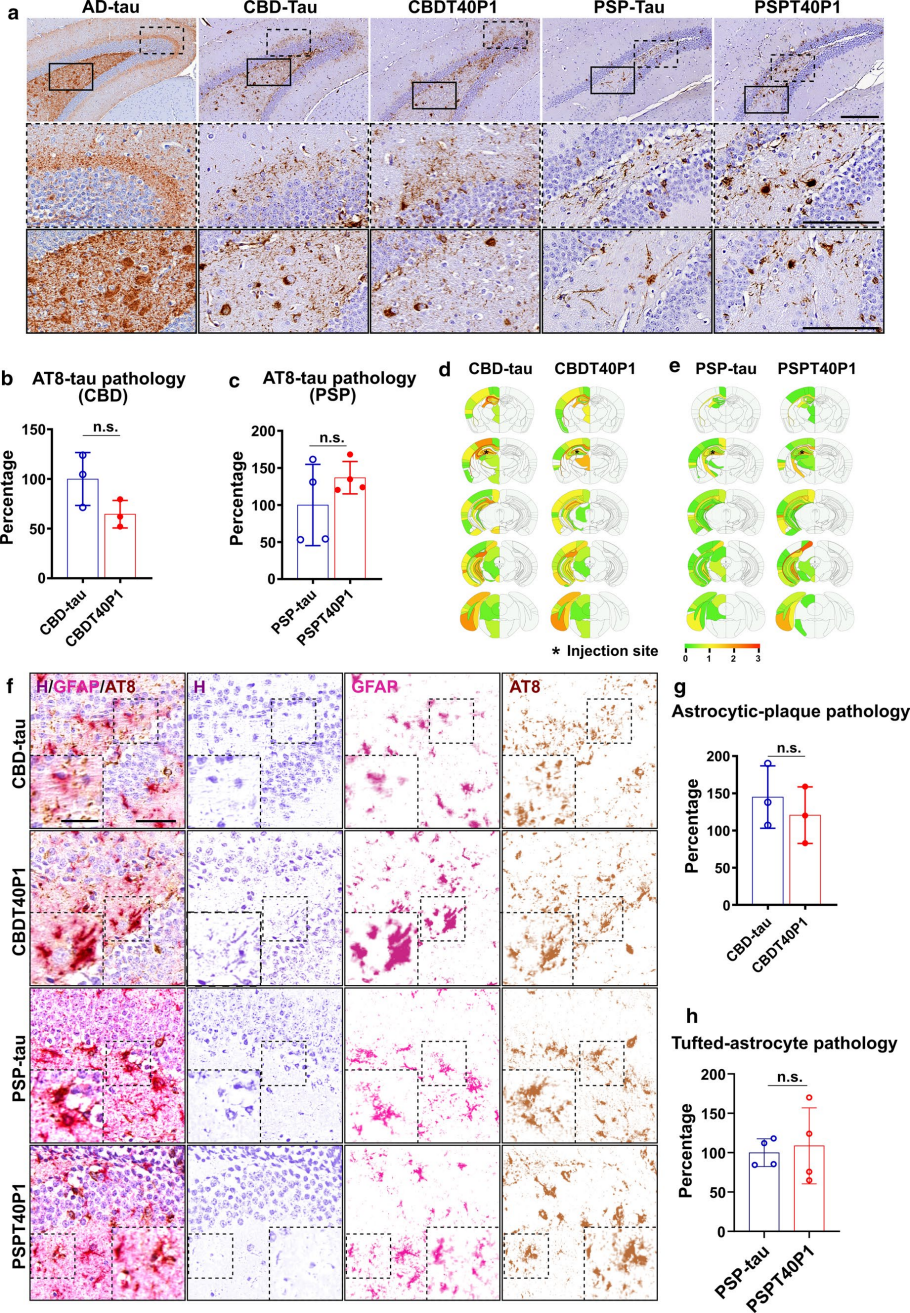
CBD and PSP strain pathogenicities are recapitulated in vivo. **a** IHC of AD-tau, CBD-tau, CBDT40P1, PSP-tau, and PSPT40P1-injected 6htau mouse brain sections show NFTs and astrocytic tau pathologies visualized using the AT8 antibody; Scale bar = 250 µm and 125 µm (inset). **b** Quantification of total tau pathology from CBD-tau- and CBDT40P1-inculated 6htau mouse in a; n.s. nonsignificant; unpaired *t* test; *n* = 3. **c** Quantification of total tau pathology from PSP-tau- and PSPT40P1-inculated 6htau mouse in a; n.s. nonsignificant; unpaired *t* test; *n* = 4. **d** Heat map shows the distribution of total tau pathology in the CBD-tau- and CBDT40P-injected 6hTau mouse brains; *n* = 3. **e** Heat map shows the distribution of total tau pathology in the PSP-tau- and PSPT40P1-injected 6hTau mouse brains; *n* = 4. **f** Double-IHC of CBD-tau, CBDT40P1, PSP-tau, and PSPT40P1-injected 6htau mouse brain reveals astrocytic plaques (AT8) and astrocytes (GFAP) in the CBD-tau- and CBDT40P1-injected 6hTau mouse brains; brain sections were costained with an anti-GFAP antibody, anti-phospho-tau antibody (AT8) and hematoxylin (H); colors were deconvoluted to show their relative distributions; insets highlight astrocytic plaques and tufted astrocytes; scale bar = 100 µm and 50 µm (inset). **g** Quantification of astrocytic-plaque pathology in CBD-tau- and CBDT40P1-injected 6htau mouse brains; n.s. nonsignificant; unpaired *t* test; *n* = 3. **h** Quantification of tufted astrocyte pathology in PSP-tau- and PSPT40P1-injected 6htau mouse brains; n.s. nonsignificant; unpaired *t* test; *n* = 4

**Fig. 8:**
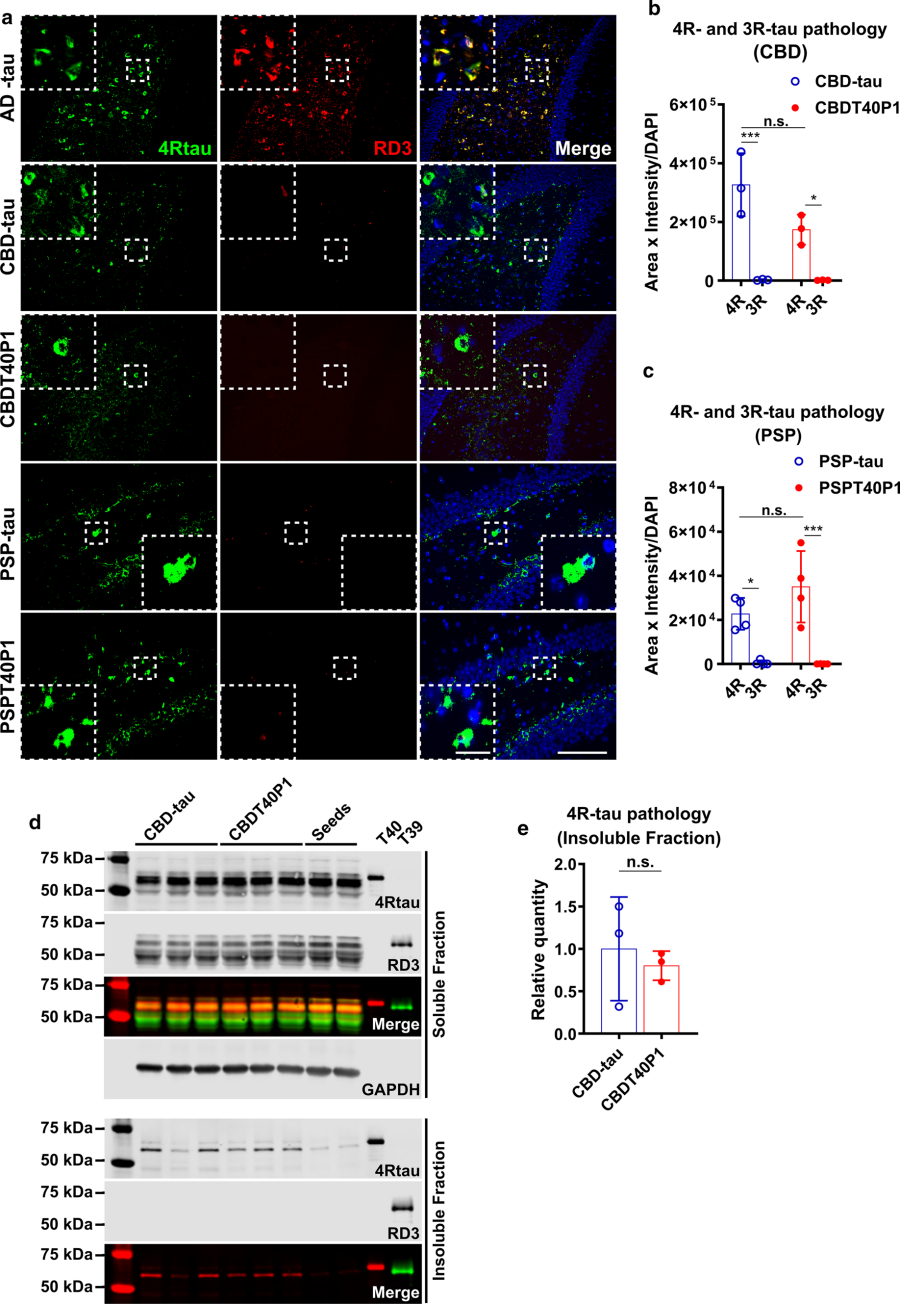
Strain-dependent isoform recruitment abilities are conserved in CBDT40P1 and PSPT40P1. **a** IF of AD-tau-, CBD-tau-, CBDT40P1-, PSP-, and PSPT40P1-injected 6hTau mouse brain sections costained with 4RTau antibody for 4R-tau and RD3 antibodies for 3R-tau; Scale bar = 100 µm and 20 µm (inset). **b** Quantification of 4R and 3R-tau pathology from CBD- and CBDT40P1-injected 6hTau mouse brain sections; *P < 0.05, ***P < 0.001; two-way ANOVA followed by Tukey post hoc test; *n* = 3. **c** Quantification of 4R and 3R-tau pathology from PSP- and PSPT40P1-injected 6hTau mouse brain sections; *P < 0.05, ***P < 0.001; two-way ANOVA followed by Tukey post hoc test; *n* = 4. **d** Immunoblots of Triton-soluble and sarkosyl-insoluble fractions of CBD-tau- and CBDT40P1-injected 6hTau mouse brains; Blots were probed with RD3 and 4Rtau antibodies for tau isoforms, and GAPDH was used as an internal loading control; **e** quantification of **d**; n.s. nonsignificant; unpaired *t* test; *n* = 3.

To examine the in vivo pathogenicity of CBDT40P1 and PSPT40P1, we performed intracerebral injections of CBDT40P1 and PSPT40P1 in 6hTau mice. For comparison, we also injected a cohort of 6hTau mice using patient-derived CBD-tau and PSP-tau. By adjusting the injected dose to account for incomplete CBDT40P1 fibrillization, the CBDT40P1-injected mouse brains exhibit comparable amounts of total tau pathology when compared with CBD-tau-injected mouse brains (Fig. 7a and b). Astrocytic plaques are one of the key diagnostic features of CBD. Both CBDT40P1 and CBD-tau induced potent astrocytic plaque pathology that is morphologically distinct and specific to CBD (Fig. 7a and f). Our quantitative analysis indicates that the total number of astrocytic plaques is statistically equivalent in CBD-tau and CBDT40P1-injected cohorts (Fig. 7f and g). Additionally, semiquantitative heatmap analysis demonstrated that the spatial distribution of CBDT40P1-induced tau pathology is very similar to the pathology induced by CBD-tau (Fig. 7d). We probed both CBD-tau- and CBDT40P1-injected brain sections with 4Rtau and RD3 antibodies and observed predominant 4R tau pathology. Unlike AD-tau and ADT40P1, CBD strains did not induce any observable 3R tau pathology (Fig. 8a and b), which supports that the isoform recruitment activity of CBD-tau is conserved in CBDT40P1. The pathological homology of CBDT40P1- and CBD-tau-injected cohorts was recapitulated by biochemical analysis of injected brain tissue (Fig. 8d and e). The data suggest that CBD-tau-specific pathogenicity was maintained in CBDT40P1 following the in vitro seeding reaction.

To assess the amplification of PSP-tau, we modified our experimental approach to adjust for the relatively low yield and purity of the extraction. Having demonstrated that the insoluble fraction of ADT40P1 shows comparable activity to AD-tau (Fig. 4c), we sought to determine whether this phenomenon could be reproduced in the PSP-tau strain. Two cohorts of 6hTau mice were injected with either the insoluble pellet fraction of PSPT40P1 or a tau concentration-matched PSP-tau control. After a post-injection interval of 3 months, both PSP-tau and pelleted PSPT40P1 induced morphologically identical neurofibrillary tangles and tufted astrocytes (Fig. 7a and f), which are unique pathological features observed in PSP patient brains [16, 30, 31]. Comparable amount of AT8-positve tau pathology (Fig. 7a and c) and tufted astrocytes (Fig. 7f and h) was found in the control and experimental groups, indicating that, like ADT40P1, the insoluble tau in PSPT40P1 accounts for its pathogenic activity. Like CBD-tau, the PSP-tau strain induced primarily 4R-tau pathology (Fig. 8a and c), indicating that the isoform recruitment ability of PSP-tau was conserved during the amplification reaction.

In sum, the in vivo data are consistent with the in vitro observations that the amplified material in CBDT40P1 and PSPT40P1 is capable of inducing pathology that are morphologically and spatially homologous to the pathology induced by the human-derived CBD and PSP-tau seeds, respectively, suggesting the strain-specific pathogenic features were conserved during the amplification procedure.

## Discussion

In the present study, we demonstrated that patient-derived tau (AD-tau, CBD-tau and PSP-tau lysates) can seed monomeric recombinant T40 in vitro, resulting in strain-dependent amplification of the pathogenic tau conformers. The amplified tau conformers potently induce strain-dependent tau pathology in both cell and animal models without requiring the overexpression of WT or mutant tau. In vitro assays and models demonstrate that the amplified material recapitulates the pathogenic features of its seeds, including insolubility, filamentous ultrastructure, and intraneuronal distribution. In vivo models demonstrate that intracerebral injection of amplified materials induces tau pathology with strain-specific, morphological and spatial–temporal features, including specific isoform recruitment and total tau-burden. This study provides the first evidence that the strain-specific pathogenic features of human-derived tau aggregates can be conferred to monomeric tau during an in vitro amplification reaction.

Previous in vitro tau seeding reactions used tau fragments that only contained the microtubule-binding domains [9] or used aggregation-prone mutant tau as the substrate to promote tau fibrillization in the reaction [5, 39]. These studies provided an important foundation for the in vitro fibrillization of tau using brain-derived tau seeds extracted from pathologically diagnosed tauopathy cases. The real-time quaking-induced conversion (RT-QUIC) and protein misfolding cyclic amplification (PMCA) assays demonstrated an increase in beta-sheet fibrillization, as measured by the huge increase (10^7^- to 10^10^-fold) in fluorescent signal of a beta-sheet binding compound [24, 29]. However, past attempts at in vitro fibrillization yielded synthetic tau strains with highly variable bioactivities and fidelity and several caveats make these fibrillization reactions unsuitable for authentic, disease-relevant amplification of tau seeds. First, these reactions used polyanionic inducers, which lead to the formation of nonspecific, preformed tau fibrils and contributed to the bioactive variability of the assembled fibrils. Second, the use of mutant tau, which has been shown to be especially prone to aggregation, also interferes with the properties of the fibrils [36]. Third, the thioflavin-based fluorescent signal has been shown to correlate poorly with the biological activities of tau conformers [1]. The bioactivity of the amplified material from previous studies remains largely unknown, and so far there is no convincing evidence supporting that the pathogenicity of tau strains could be amplified and maintained in multiple cycles.

In this study, we chose to use T40, a full-length 2N4R tau isoform, as the monomeric substrate because it contains all the domains of mature tau in the adult human brain. Importantly, T40 is the common tau isoform present in AD-tau, CBD-tau and PSP-tau. The single-cycle fibrillization reaction did not include any known polyanionic inducers or cofactors to eliminate spontaneous de novo tau fibrillization. Additionally, our use of physiological in vitro and in vivo models with endogenous mouse tau expression provides the necessary resolution to differentiate between the pathogenic conformers. These experimental systems allow us to generate bioactive tau strains in vitro and accurately assess their strain-specific pathogenicities.

Of note, we did not observe evidence that the amplified fibrils are active in secondary in vitro amplification reactions. Two hypotheses could explain the inertia of the amplified products in subsequent in vitro amplification reactions. One hypothesis is that the initiation of tau aggregation in human and mouse neurons is driven by pathogenic cofactors, which are needed to induce the soluble tau monomers into an intermediate state that allows them to interact with the seeds. While these cofactors could be present in the disease brain lysates during the first cycle of amplification, as well as in the mouse brains and in the cultured neurons, they may get diluted in secondary cycles of in vitro amplification. Previous studies have also hypothesized that cofactors could affect tau behavior in in vitro reactions [6, 38]. This hypothesis is also consistent with the observation that in the PMCA and QUIC assays, polyanionic factors must be present for multiple cycles of amplifications. To address this question, human tau seeds with higher purity will be needed to further assess the role of these cofactors in tau pathogenesis.

The second hypothesis is that posttranslational modifications of human tau fibrils enable the human-derived tau seeds to interact with recombinant tau and induce fibrillization. The T40 monomers, and thus, the resulting amplified material, lack the active sites necessary for successive seeding activity due to the absence of posttranslational modification. In human disease brains, tau fibrils are known to be extensively posttranslationally modified and, according to this hypothesis, these posttranslational modifications may be necessary for the fibrils to interact with tau monomers. In the first round of in vitro amplification, the T40 monomers can interact with human-derived tau seeds to form fibrils. However, during this initial amplification reaction, the active tau seeds become saturated with T40 fibrils, which lack posttranslational modifications. Thus, the resulting amplified fibrils are inert and can no longer induce fibrillization in vitro. Despite their inertia in subsequent in vitro amplification reactions, the amplified materials are potent in neurons and mouse brains because the amplified fibrils are reactivated by posttranslational modifications following endocytosis. This hypothesis is consistent with our observation that, at the endpoint of the amplification reaction, some of the T40 monomers remain unfibrillized (Supplementary Fig. 1a, online resource). Experimental evidence of posttranslational modifications and their effect on the fibrillization of tau will be essential to further address this hypothesis.

The quality of the extracted tau seeds is crucial for the in vitro amplification reactions. It is well documented that tau can bind to anions or lipids in the cells, which could lead to variability in quantifying the amount and/or activities of the amplified material. To enrich for brain-derived tau seeds in our study, we chose tauopathy cases at late stages of the disease where tau-burden is high in many brain regions. The presence of copathology in a primary tauopathy case may lead to the generation of heterogeneous tau seeds, so the extracted brain regions were also histochemically screened for other proteinopathic comorbidities, such as a-synuclein and TDP-43 inclusions. Brain tissue containing comorbid pathology was not selected for extraction, which enabled a higher yield and greater enrichment of high-quality tau seeds. Furthermore, using this approach to human tissue selection, we were able to experimentally determine that tau seeds isolated from different individuals with the same type of tauopathy are comparable in terms of their biochemical properties as well as pathogenicity in cell and mouse models. Despite our thorough screening process, we cannot exclude the possible existence of subspecies of tau seeds that could be underrepresented by the enrichment procedure. A systematic investigation of different brain regions, disease courses, and cell types will be needed to address the possible heterogeneity of human-derived tau seeds.

Additionally, the CBD and PSP lysates were much lower in their concentration and purities compared to AD-tau, which is due to a lower tau-burden in CBD and PSP patient brains. Despite the relatively low yield and quality of the CBD and PSP lysates, we did observe a comparable increase in the activity of the amplified material, which was consistently a two- to threefold increase in activity compared to the seeds control across the different tau strains. The consistency of amplification across different lysates strongly indicates that tau seeds, but not other insoluble molecules in the lysates, are recruiting the recombinant tau monomers. However, we have not yet to rule out the possibility that insoluble substances in the lysates will interact with tau and cause nonspecific aggregation of tau monomers. Nonetheless, to effectively increase the amplification efficiency further, a higher purity of tau seeds may be necessary.

Additionally, our results indicate that multiple isoforms of tau are not required for the faithful fibrillization of tau in vitro, but we have yet to explore whether the presence of multiple tau isoforms could promote fibrillization in the seeding reaction. In a previous study, we tested seeding reactions using recombinant human tau with a 1:1 ratio of 3R and 4R tau isoforms and found no significant increase compared to the single isoform reactions [14], yet we cannot rule out the possibility that a different combination of monomers could lead to different outcomes of the seeding reaction. Further experiments to address these possible variables could also optimize the efficiency of the fibrillization reaction.

In human brains, the transmission of tau pathology occurs in mature neurons with intact neuroanatomical connectomes. However, since pathological tau is also found in oligodendrocytes and astrocytes, these glial cells could play an important role in the development of tau pathology and its transmission in the disease state. Thus, it is essential to use intact animals for in vivo studies of tau pathology transmission. In this study, we confirmed the pathogenicity of ADT40P1 in vivo using both 5xFAD transgenic mice and WT mice. The 5xFAD mice overexpress human mutant APP and PSEN1, which leads to the accumulation of amyloid-beta plaques in the adult mouse brain [32]. However, both the 5xFAD and WT models express endogenous mouse tau at physiological concentrations and do not develop tau pathology without injection of human tau seeds. Synthetic tau pffs are not capable of inducing a significant amount of tau pathology in either the 5xFAD mouse or WT mouse brains [14]. Intracerebral injection of human tau seeds into 5xFAD mice (at least 4 months of age) will lead to the formation of neuritic plaque tau pathology at the one-month post-injection interval [15]. Thus, the 5xFAD mice are a robust and reliable model for quantitative measurement of tau pathology with the resolution required to differentiate human tau seeds from artificially assembled tau pffs.

In this study, the increased tau pathology observed in 5xFAD mice following intracerebral injection of ADT40P1 versus a 10% AD-tau seeds control is consistent with the in vitro data from the neuron culture assay, confirming that ADT40P1 is pathogenic and potently induces tau pathology in vivo. ADT40P1 also induced a significant amount of tau pathology in WT mice, further validating its disease-relevant pathogenicity. ADT40P1 induced neuritic plaque tau pathology indistinguishable from that induced by extracted AD-tau, suggesting that ADT40P1 could be used in lieu of AD-tau, which is a very limited resource, for further in vivo studies of tau pathogenesis.

Prior studies have demonstrated that different tau strains are composed of different tau isoforms [37]; however, there has been no direct evidence demonstrating that the tau isoform composition in the fibrils dictates strain-specific bioactivity. We hypothesize that the structural conformation of the fibrils, rather than the isoform composition, determines the bioactivity of different tau strains. To demonstrate this hypothesis, we interrogated the activity of ADT40P1 and CBDT40P1 in 6hTau transgenic mice as an additional in vivo model. The 6hTau mice express all six isoforms of human WT tau on a null mouse tau background, mimicking the isoform expression patterns in the human adult brain and providing a human-like tauopathy model to elucidate the isoform recruitment of different tau strains [27]. With WT human tau overexpression, the 6hTau mice are free of tau pathology throughout their lifespan. However, intracerebral injection of human tau seeds in 6hTau mice induced strain-specific tau pathology that correlated with the tau isoform composition of the seeds [45]. We normalized the total tau pathology by scaling the dose of the injected material so that we could perform a direct comparison of different tau isoform components in the induced tau aggregates. We observed that the tau aggregates in ADT40P1- and AD-tau-injected cohorts had the same ratios of 3R to 4R tau isoforms despite the fact that the ADT40P1 fibrils are primarily composed of 4R tau. CBDT40P1 and CBD-tau injected cohorts had primarily 4R tau aggregates. Together with the PK digestion data, these findings indicate that the pathogenicity of amplified material was inherited through the transmission of the abnormal tau conformation, rather than the isoform composition. The data also suggest that the isoform recruitment is a feature of strain-specific bioactivity, rather than a causal agent for the formation of different tau seeds. Here, we demonstrated that a single tau isoform converted by different tau strains into a pathological conformation is sufficient to recapitulate the signature bioactivity, including strain-specific isoform recruitment, of pathological tau in vivo.

Our study demonstrates that the pathogenicity and biophysical properties of human-derived tau seeds are conferred to and conserved in the products of in vitro amplification reactions. These findings not only demonstrate an experimental framework for understanding disease-specific tau fibrillization, but are also a proof-of-concept that, under proper conditions, in vitro amplification reactions yield pathogenic material that recapitulates the bioactivity of human-derived tau seeds that can dramatically increase the availability of pathogenic tau material for future studies of tau pathogenesis.

## Supplementary Information

Below is the link to the electronic supplementary material.

Supplementary file1 Supplemental Figure 1 Protease inhibitors (PIs) effectively stop degradation of T40 in the seeding reaction. a Immunoblot of sedimentation assay for from in vitro seeding reactions with/without protease inhibitors (PI); S supernatant; P pellet; 17025 total tau antibody; PHF1 phospho-tau antibody; OE overexposed; samples were fractionated under 100,000 g for 30 min. Supernatant and pellet represent soluble and insoluble fractions of the sample. b Quantification of pellet fraction (indicated by arrowhead) in ADT40P1with/without PI from a; ** P < 0.01, unpaired t test; n=4. c ICC of WT neurons treated with products from in vitro seeding reactions with/without protease inhibitors; scale bar=100 µm. d Quantification of c; T49-positive area was normalized to MAP2 area; 100% seeds activity level was set as 100%; n.s. nonsignificant 10% seeds vs 10% seeds+PI vs T40+PI, ** P < 0.01 ADT40P1-PI vs ADT40P1+PI, ADT40P1+PI vs 10% seeds+PI, ADT40P1+PI vs T40+PI, one-way ANOVA followed by Tukey post hoc test; n=4. (TIF 101902 KB)

Supplementary file2 Supplemental Figure 2 Titration of protease inhibitors for the in vitro seeding reactions. a ICC of neurons treated with products from in vitro amplification reactions; tau pathology was visualized using T49 mouse tau-specific antibody; addition of a protease inhibitor in the reaction resulted in greatest activity increase; ADT40P1 with different concentrations of protease inhibitor in the reactions shows no toxicity; 100% seeds activity level was set as 100%; scale bar=100 µm. b Quantification of T49-labeled mouse tau pathology in a; * P < 0.05 10% seeds vs ADT40P1 PI (1:200), *** P < 0.001 10% seeds vs ADT40P1 PI (1:50) or ADT40P1 PI (1:100), one-way ANOVA followed by Tukey post hoc test; n=4. c Quantification of T49-mouse tau pathology in a; n.s. nonsignificant; one-way ANOVA followed by Tukey post hoc test; n=4. (TIF 41159 KB)

Supplementary file3 Supplemental Figure 3 CBL-seeded T40 reaction and heparin-induced T40 pffs were impotent on WT neurons. ICC of neurons treated with products from in vitro amplification reactions or AD-tau; tau pathology was visualized using the T49 antibody; no pathology was found in PBS, CBL-seeded T40P1 (CBLT40P1), or hep-T40-treated cells; scale bar=100 µm. (TIF 25488 KB)

Supplementary file4 Supplemental Figure 4 Heparin-induced tau pffs (hep-T40) were inactive and did not induce tau pathology in 5xFAD mice. IHC of 6-month-old 5xFAD mouse brains following inoculation of the mouse brains with 2 μg of hep-T40 for 1-month post-injection. AT8 antibody was used to visualize tau pathology; no neuritic tau pathology was found in the 5xFAD mouse brains with hep-T40 inoculation; upper panel scale bar=200 µm; lower panel scale bar=100 µm. (TIF 15060 KB)

Supplementary file5 Supplementary Figure 5 ADT40P1 is pathogenic in WT mice. a IHC of WT mouse brains intracerebrally injected with ADT401P1, 100% seeds or 10% seeds at 6-month post-injection; phospho-tau is visualized using AT8. Ipsi-HP ipsilateral hippocampus; Con-HP contralateral hippocampus; EC entorhinal cortex; scale bar=250 µm and 125 µm (inset). b Quantification of AT8-tau positive pathology from a; 100% seeding activity level was set as 100%; * P<0.05, ** P<0.001; one-way ANOVA followed by Tukey post hoc test; n=3. c Heat map shows the distribution of AT8-positive total tau pathology and NFTs in 100% seeds control and ADT40P1 group; n=3. (TIF 101887 KB)

Supplementary file6 Supplemental Figure 6 A low ratio of AD-tau to T40 monomer is sufficient to seed T40 in vitro. a IHC of tau pathology in WT neurons treated with ADT40P1* (ADT40P1*=1% AD-tau + 99% T40, (after shaking for 14-21 days) or AD-tau. R2295M visualizes mouse tau pathology; AT8 visualizes phospho-tau; DAPI visualizes cell nuclei; scale bar=100 µm. b Transmission EM of sonicated (son) AD-tau (seeds for reactions), ADT40P1* and T40 shaken for 21 days; scale bar=100 nm. c IHC of 6-month-old 5xFAD mouse brains injected with AD-tau or ADT40P1*; mice were killed at 1-month post-injection, and brain sections were stained by AT8 antibody; upper panel scale bar=200 µm; lower panel scale bar=100 µm. d Immunoblots of fractionated lysates extracted from AD-tau and ADT40P1*-injected 5xFAD mouse hippocampi; total tau was visualized using K9JA antibody; phospho-tau was visualized using PHF1 antibody; mouse tau was visualized using R2295M antibody; GAPDH was used as a loading control. e Quantification of R2295M-positive insoluble mouse tau from a; R2295M-positive area was normalized to MAP2 area; 100 % AD-tau seeding activity was set as 100 %; ** P < 0.01, unpaired t test; n=4. f Quantification of AT8 tau pathology from c; 100 % AD-tau seeding activity was set as 100%; ** P < 0.01, unpaired t test; n=6. g Quantification of d; 100 % AD-tau seeds level was set as 100 %; * P < 0.05, *** P < 0.001, unpaired t test; n=3. (TIF 101972 KB)

Supplementary file7 Antibodies used in the study (XLSX 12 KB)
