# Supplementary figures and images for: In vitro amplification of pathogenic tau conserves disease-specific bioactive characteristics
Source: Acta Neuropathol. 2021 Jan 1;141(2):193–215. doi: 10.1007/s00401-020-02253-4 (PMC7847465; doi:10.1007/s00401-020-02253-4)

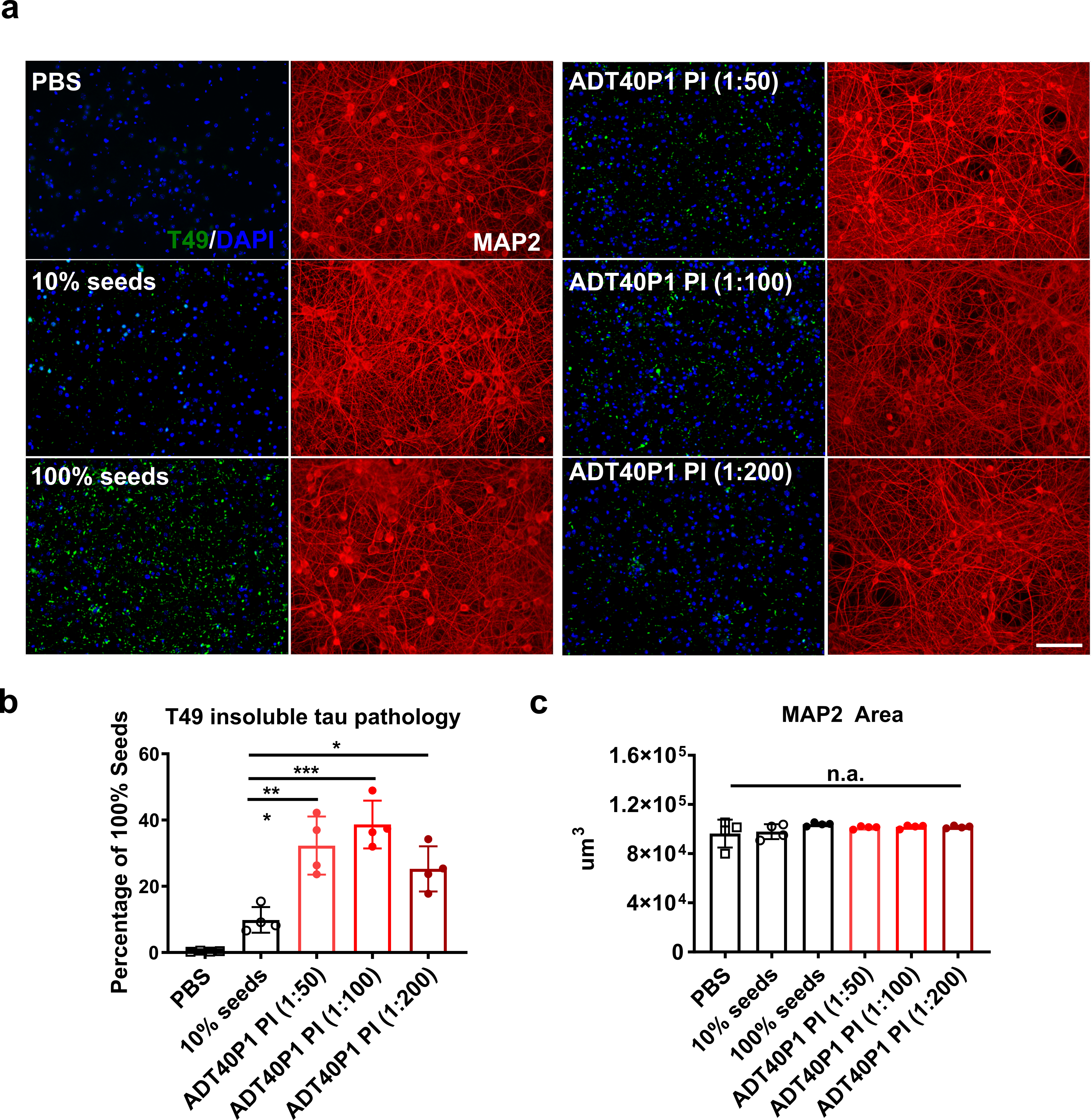

Supplement: Supplementary file 2 — Supplementary file2 Supplemental Figure 2 Titration of protease inhibitors for the in vitro seeding reactions. a ICC of neurons treated with products from in vitro amplification reactions; tau pathology was visualized using T49 mouse tau-specific antibody; addition of a protease inhibitor in the reaction resulted in greatest activity increase; ADT40P1 with different concentrations of protease inhibitor in the reactions shows no toxicity; 100% seeds activity level was set as 100%; scale bar=100 µm. b Quantification of T49-labeled mouse tau pathology in a; * P < 0.05 10% seeds vs ADT40P1 PI (1:200), *** P < 0.001 10% seeds vs ADT40P1 PI (1:50) or ADT40P1 PI (1:100), one-way ANOVA followed by Tukey post hoc test; n=4. c Quantification of T49-mouse tau pathology in a; n.s. nonsignificant; one-way ANOVA followed by Tukey post hoc test; n=4. (TIF 41159 KB) [file 401_2020_2253_MOESM2_ESM.tif]

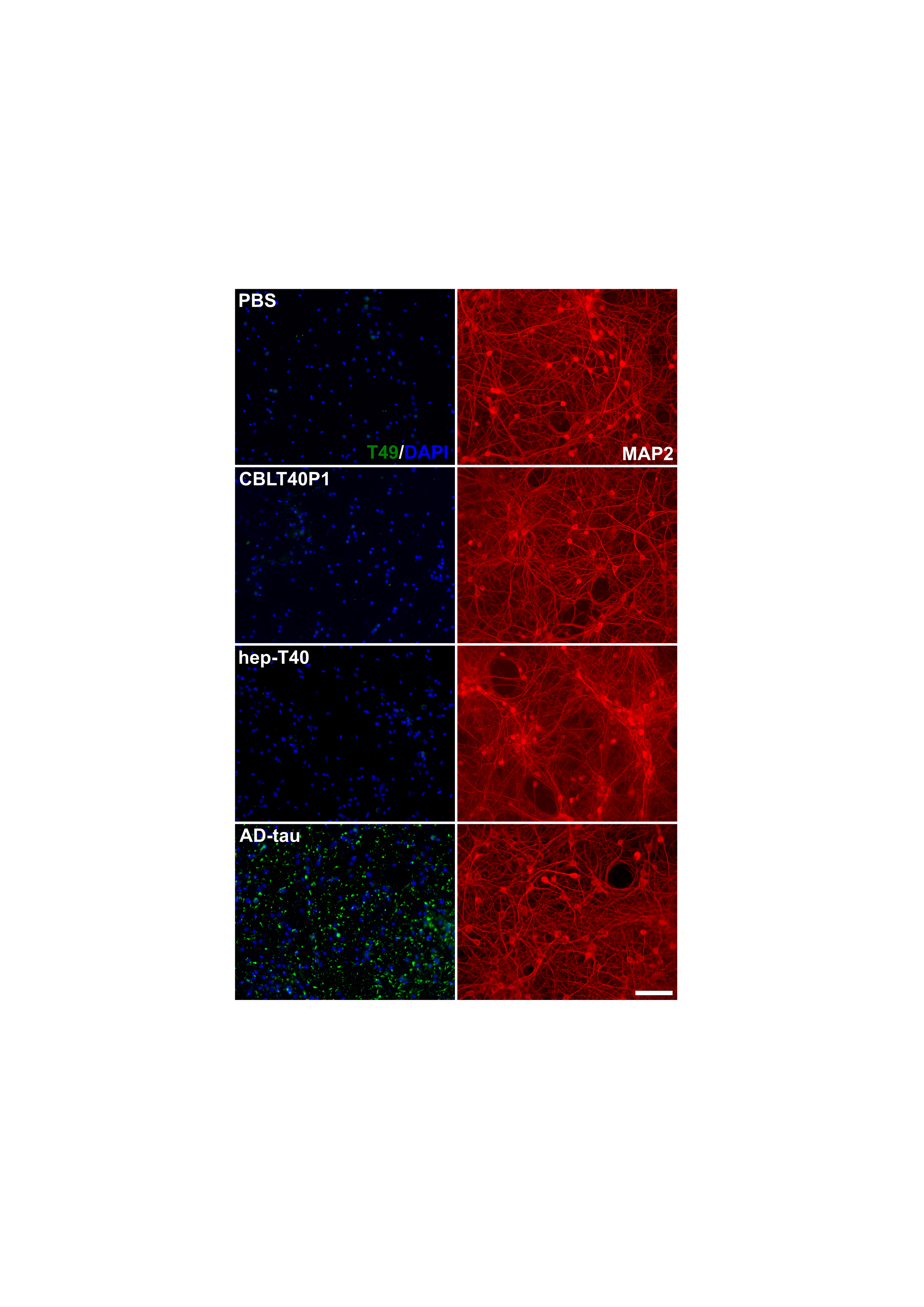

Supplement: Supplementary file 3 — Supplementary file3 Supplemental Figure 3 CBL-seeded T40 reaction and heparin-induced T40 pffs were impotent on WT neurons. ICC of neurons treated with products from in vitro amplification reactions or AD-tau; tau pathology was visualized using the T49 antibody; no pathology was found in PBS, CBL-seeded T40P1 (CBLT40P1), or hep-T40-treated cells; scale bar=100 µm. (TIF 25488 KB) [file 401_2020_2253_MOESM3_ESM.tif]

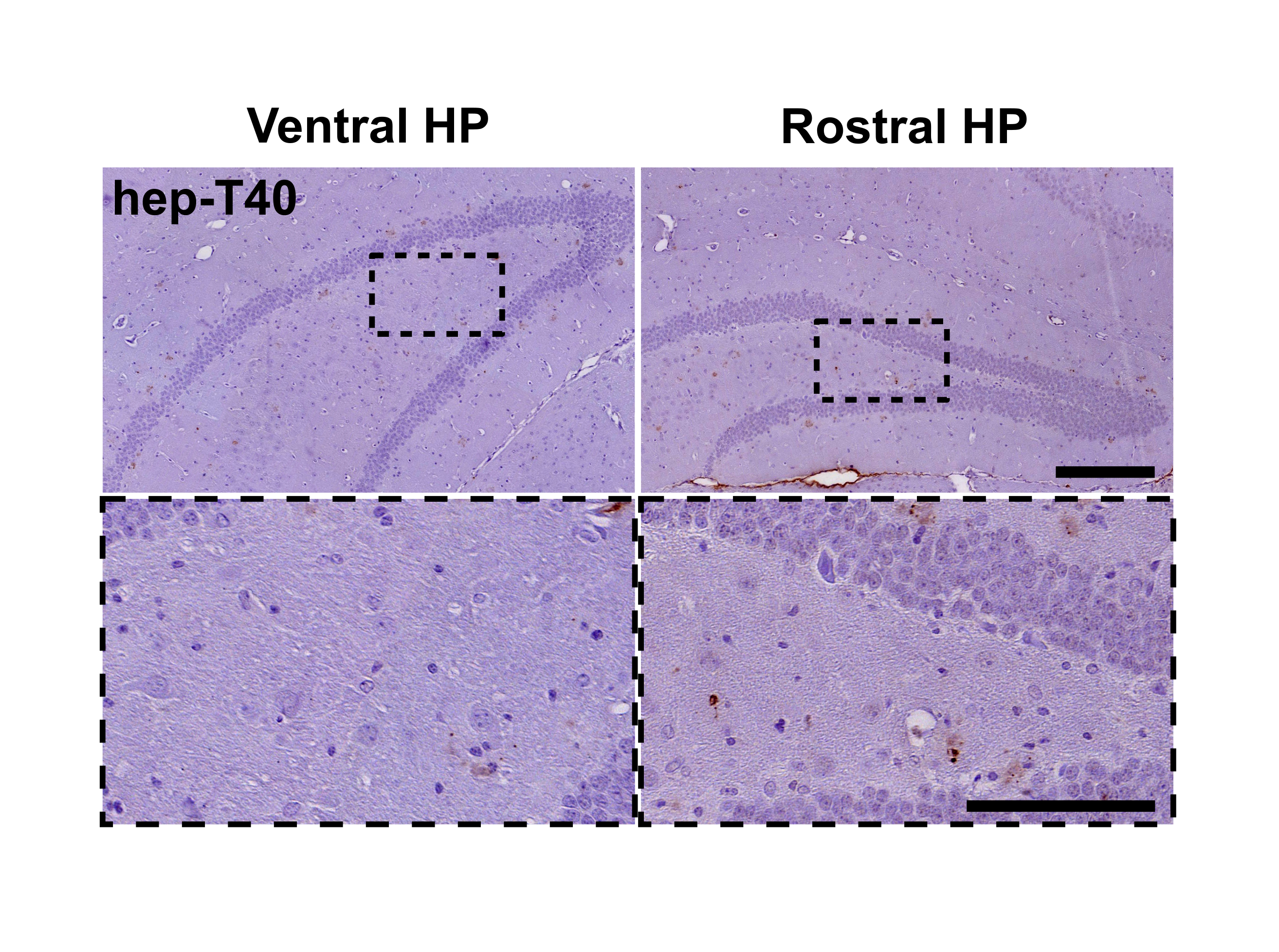

Supplement: Supplementary file 4 — Supplementary file4 Supplemental Figure 4 Heparin-induced tau pffs (hep-T40) were inactive and did not induce tau pathology in 5xFAD mice. IHC of 6-month-old 5xFAD mouse brains following inoculation of the mouse brains with 2 μg of hep-T40 for 1-month post-injection. AT8 antibody was used to visualize tau pathology; no neuritic tau pathology was found in the 5xFAD mouse brains with hep-T40 inoculation; upper panel scale bar=200 µm; lower panel scale bar=100 µm. (TIF 15060 KB) [file 401_2020_2253_MOESM4_ESM.tif]
